# Dynamic Coupling between Tom22 Motions and Tom40 Pore Dynamics Modulates Ion Transport in the Mitochondrial TOM Complex

**DOI:** 10.1021/acs.jcim.5c01761

**Published:** 2025-10-31

**Authors:** Abhishek Acharya, Stephan Nussberger, Shuo Wang, Ulrich Kleinekathöfer

**Affiliations:** † School of Sciences, 84498 Constructor University, Campus Ring 1, 28759 Bremen, Germany; ‡ Department of Biophysics, Institute of Biomaterials and Biomolecular Systems, University of Stuttgart, 70569 Stuttgart, Germany; ¶ Department of Bionanoscience, Kavli Institute of Nanoscience Delft, Delft University of Technology, 2628 CJ Delft, The Netherlands

## Abstract

Mitochondria rely on the efficient import of proteins to maintain their functions and regenerate. The translocase of the outer mitochondrial membrane (TOM) complex serves as the primary entry point for the import of mitochondrial proteins. Previous studies have established Tom22 as a multifunctional subunit within the complex and reported mechanosensitive gating-like behavior of the TOM complex. In this study, all-atom molecular dynamics simulations of the TOM core complex reveal large motions of the Tom22 helices that are coupled to global structural rearrangements within the complex, particularly with the α2 helix within the Tom40 pore subunit. Microseconds-long simulations with restraints on the Tom22 helices yield an alternative conformation of the α2 helix that is associated with a reduced ion permeability. The outcome corroborates previous experimental results that reported a reduction in calcium ion flux for transiently stalled TOM complexes. These findings provide a molecular view of a mechanism by which Tom22 modulates the pore architecture of Tom40 and regulates permeability, thus linking the receptor dynamics to the functional control of the mitochondrial protein import.

## Graphical abstract

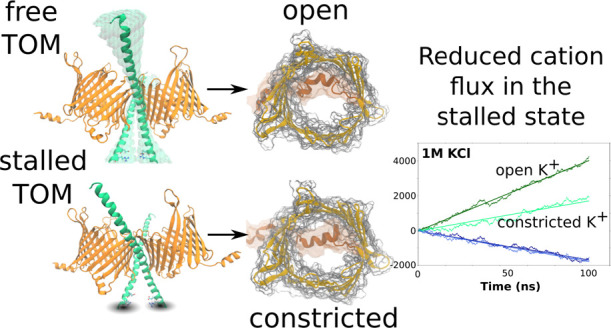

## Introduction

The mitochondrial TOM complex is of paramount importance for the import of nuclear-encoded mitochondrial proteins.^1,2^ It resides in the outer mitochondrial membrane (OMM) and facilitates the transport of mitochondrial precursor proteins from the cytosol across the membrane through its pore subunits. The majority of the precursor proteins (≈60%) have an N-terminal cleavable presequence containing a mitochondrial targeting signal that enables the selective capture and transport by the TOM machinery. Following import through TOM, the complex engages with additional protein factors, including the intermembrane space chaperones, TIM22, TIM23, and SAM, to guide the preproteins to their final mitochondrial destinations.^3–9^

Recently, high-resolution structures of *Neurospora crassa*, *Saccharomyces cerevisiae* , *Drosophila melanogaster* , and human TOM complexes (Figure 1A) have been determined by cryo-electron microscopy (cryo-EM).^10–14^ All of them exhibit at least a dimeric core structure, the so-called TOM core complex (TOM-CC), where each asymmetric unit consists of a 19-stranded β-barrel pore (Tom40) associated with four transmembrane (TM) α-helical transmembrane proteins (Tom5, Tom6, Tom7 and Tom22), although higher order assemblies have also been reported in most cases.^10–19^ Additional mitochondrial subunits, Tom20 and Tom70, form the TOM holo complex wherein each subunit is anchored to the membrane by a single α-helical TM segment.^1^ Tom20 and often Tom70 are involved in the initial capture of precursor proteins from the cytoplasm and assist in the transfer to Tom22 and the Tom40 pore.^20–23^ Tom20 interacts with Tom22 and is positioned close to Tom40.^14,24–27^ The details of Tom70 interaction with the TOM-CC, however, remain to be resolved.

**Figure 1:**
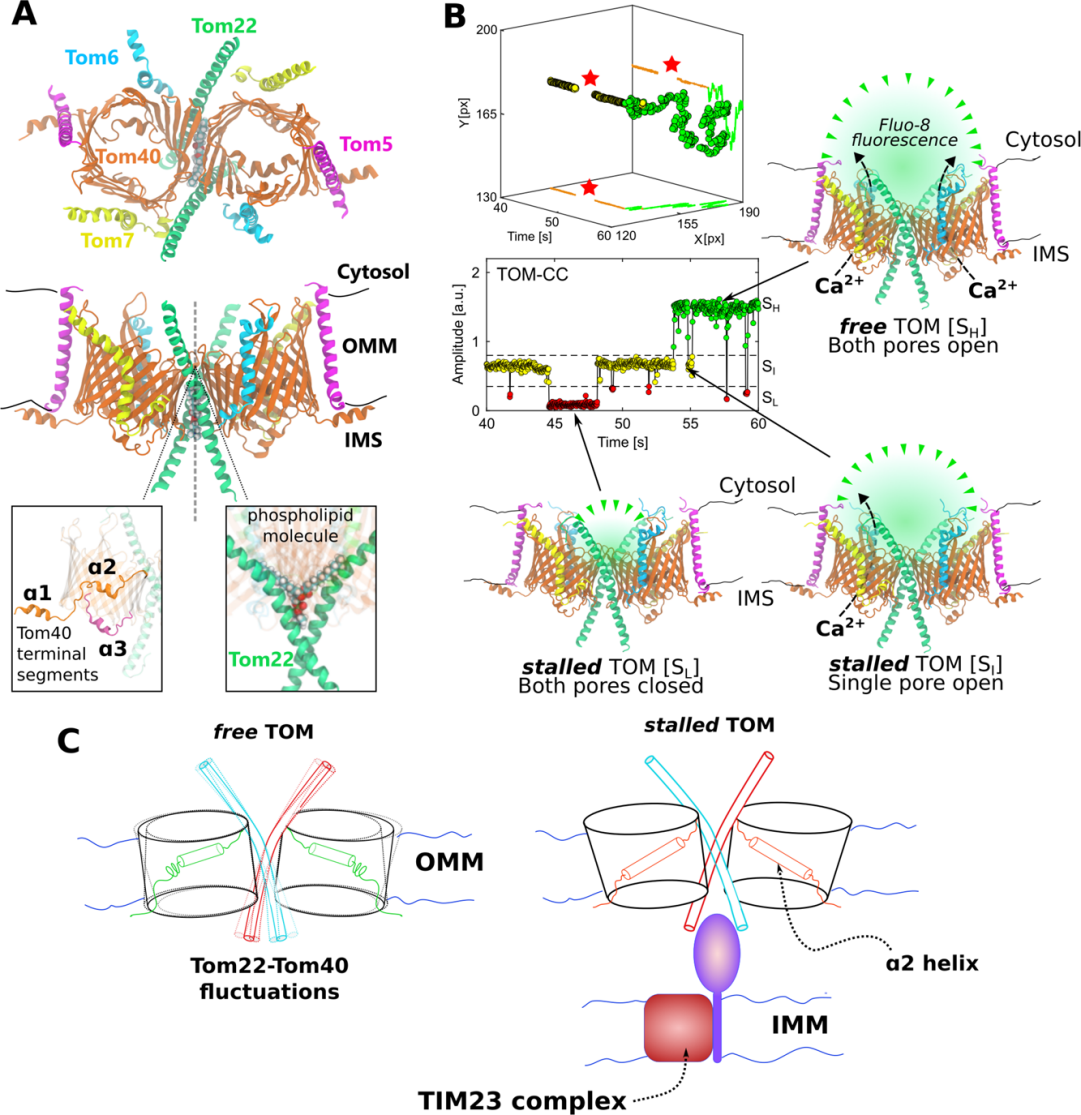
Overview of the TOM-CC structural and the functional aspects explored in this study. (A) Cryo-EM structure of the TOM-CC (PDB ID: 8B4I), shown from the cytosolic side (top) and in membrane cross-section (middle). Close-up views highlight the N- and C-terminal domains of Tom40 (left) and the Tom40–Tom22 dimer interface (right). (B) Optical detection of Ca^2+^ flux through the TOM-CC. The fluorescent amplitude trace (bottom) shows transitions between three conductance states: S_L_ (both pores closed), S_I_ (one pore open), and S_H_ (two pores open). The *xy*-position plot (top) illustrates the lateral mobility of the TOM complex within the membrane. The stars denote missing data points that correspond to the S_L_ state whose position is undetected due to absence of the fluorescence signal. The intensity and position plots are reproduced with permission from ref 37. Available under a CC-BY 4.0 license. Copyright 2022 Wang et al. (C) Schematic representation of model of the mechanosensitive gating behavior of TOM described in this work. The free TOM shows conformational fluctuations in the Tom22 helices (red and blue rods) and the Tom40 barrels. The stalled TOM, stabilized due to interactions with protein partners on the IMS (prominently the TIM complex), shows a prominent shift in the α2 helix at the center of the Tom40 barrel. This shift corresponds to a narrowing of the Tom40 pore.

The *N. crassa* TOM-CC cryo-EM structure features two tilted Tom40 β-barrels, bridged at the dimer interface by two Tom22 helices.^10^ The structure is similar to the VDAC channel that is also composed of 19 β-strands.^28–30^ The structural similarities reflect the common origin of these channels from bacterial ancestors.^31^ While VDAC is a highly versatile channel involved in a vast array of cellular pathways and its activity modulated by a myriad of protein partners,^32^ the Tom40 barrel have evolved toward a limited functional scope wherein it mediates the transport of preproteins within the TOM complex.^31,33^ Another contrasting property is the selectivity of the channels with the VDAC being an anion-selective pore and Tom40 being cation-selective.^34^ The evolution of pore electrostatics mirrors the functional context. The cation-selectivity of Tom40 is important for the capture and transport of positively charged mitochondrial presequences, and the anion-selectivity of VDAC are critical for the transport of ADP, ATP, and other negatively charged metabolites. VDAC, however, can also shift to a weakly cation-selective state in association with effector proteins.^35^ Both Tom40 and VDAC possess an N-terminal segment that passes through the β-barrel. In VDAC, this region is involved in the conformational shift in ion selectivity^36^ and also interacts with effector proteins.^30^ In Tom40, the N-terminal is also thought to play a functional role in the preprotein transport process. The Tom40 β-barrel accommodates a short N-terminal domain containing two α-helices (α1 and α2; Figure 1A) where α2 resides within the barrel and interacts with strands β11−β17. A third helix (α3) is located near the IMS side of the membrane.

The Tom22 helices are kinked by ∼30°, a distortion mediated by a conserved proline residue, and interact with each other at a helix tilt angle of ∼20°, extending into both the cytosol and the mitochondrial intermembrane space (IMS). The cytosolic domains of Tom22 are not interacting, whereas the IMS domains are comparatively much closer, though not interacting. Juxtaposed between the two Tom22 subunits is a single phospholipid molecule, where the acyl chains and the IMS-facing headgroup contribute to the intersubunit contacts (Figure 1A). Tom22 subunits consist of an α-helix of ∼80 Å length including a 22 Å C-terminal segment which protrudes into the IMS and a TM segment of 38 Å. The 20 Å cytosolic N-terminal segment of the helix is oriented parallel and seems to be associated with the outer membrane surface. It should be noted that the outermost N- and C-terminal regions of Tom22 are not observable in the cryo-EM structures, possibly due to local disorder. The dynamics of the two Tom22 subunits are of significant interest due to their potential role in regulating the opening/closing dynamics of the two Tom40 channels.^37,38^

While the reported cryo-EM structures combined with biochemical studies have helped uncover details of the role of the TOM apparatus in the import of precursor proteins and coordination with other import complexes,^1,34,39,40^ the underlying dynamics of the complex and the mechanism driving the protein import is less understood. The available presequence-bound structures and cross-linking studies suggest that the TOM complex is sufficient to mediate presequence transport from the cytosol to the IMS-facing side.^41^ However, it is insufficient to mediate the efficient translocation of the full preprotein on its own and requires assistance from additional proteins on the IMS side.^42^ At the same time, in the absence of any apparent driving force, the presequence translocation step through the TOM complex is particularly intriguing. Affinity-driven transport from the cytosolic receptors toward the IMS has been proposed as a possible mechanism, but the evidence for an obvious affinity gradient within the Tom40 pore is missing.^1^ A Brownian ratchet mechanism wherein thermal fluctuations of the complex enable the gradual transport of a polypeptide is another possibility.^40^ Interestingly, electrophysiological experiments on TOM-CC reconstituted into lipid bilayers have revealed voltage-sensitive gating behavior, with Tom40 exhibiting fluctuating ionic conductance and transitions between distinct substates at high voltages.^18,43–48^ Although the outer mitochondrial membrane is thought not to have a significant membrane potential that could influence TOM activity,^37^ electrophysiological analyses can still yield important clues regarding the conformational dynamics of the channel.^17,48–51^ For the TOM-CC, such studies indicated the presence of mobile structural elements within the complex that may influence the import activity, although the precise molecular details of the underlying mechanism and associated conformational states are unresolved. Recently, a new single-molecule optical approach using total internal reflection fluorescence (TIRF) microscopy, which is performed in the absence of external voltage and thus under a more physiological condition, has shed new light on the dynamics and ion-transport behavior of the TOM-CC.^37^

Observation of Ca^2+^-transport through the complex revealed three states of high, intermediate, and low ion flux (Figure 1B). Surprisingly, these states are affected by lateral protein membrane mobility. Freely moving TOM complexes exhibited high Ca^2+^-flux corresponding to the open state of the Tom40 pores, whereas immobilization of the TOM complex via the IMS domains of the Tom22 subunits led to pore closures. This has been further supported by the finding that Tom40 channels without additional subunits remained predominantly open. Since Tom22 is the only subunit able to physically interact with an immobilizing phase below the membrane (e.g., adjacent protein subunits in the IMS in mitochondria), it has been suggested to play a key regulatory role for Tom40 pore closure, possibly mediated by a mechanosensitive conformational change. Moreover, several studies have independently established Tom22 as a critical component of the TOM machinery that functions as a receptor domain,^46,52,53^ a docking site for the Tom20 receptor and overall organization of the TOM-CC dimer.^14,24^ In yeast mitochondria, studies on the TOM complex extracted from both wild-type and *Δtom22* strains suggested a possible role of Tom22 in a negative regulation of the opening and closing dynamics of the pore.^46^ In the light of the available structural and biochemical data, detailed atomistic simulations of the TOM core complex are expected to provide important hints on its overall conformational dynamics and the role of Tom22 in the functional regulation. With this objective, we have used all-atom molecular dynamics simulations to investigate the dynamical behavior of the TOM-CC to unravel the mechanistic details of the Tom22/Tom40 interaction mediating the conformational dynamics of the TOM complex, and Tom40 pore closures. Our results show that conformational motions of Tom22 are closely linked to structural rearrangements within the Tom40 pore, particularly of the intrapore helix α2 located at the narrowest region of the Tom40 barrel. These structural changes impact the ion flux and suggest a mechanism for Tom22-mediated regulation of the import function of TOM.

## Material and Methods

### System Preparation

The coordinates for the *N. crassa* TOM-CC were obtained from the cryo-EM structure deposited at the Protein Data Bank (PDB ID: 8B4I).^14^ While the core structure of the subunits that form the transmembrane part of the structure is well resolved, the disordered terminal regions at the cytosolic and the IMS side are missing. For instance, a 60-residue N-terminal segment and about 30 residues at the C-terminal of the Tom22 helix is disordered. To reduce the system complexity, we chose to leave out the disordered parts of the Tom22 subunits with the assumption that these regions are largely important for the substrate capture and interactions with accessory proteins, and their absence does not substantially affect the inherent dynamics and ion transport properties of the TOM-CC. With the same assumption, some part of the disordered N-terminal regions of Tom40 (1–24), Tom5 (1–4) and Tom6(1–4) were excluded. The system with the TOM-CC containing the remaining integral segments, embedded in a symmetric DPhPC (1,2-diphytanoyl-*sn*-glycero-3-phosphocholine) lipid membrane inside a solvated box, was modeled initially with the help of CHARMM-GUI.^54–56^ Although, the native mitochondrial membrane is composed of mixture of phosphatidylcholine, phosphatidylethanolamine and additional lipid components, we used DPhPC that were used for the single-channel optical ion flux experiments.^37^ The structure obtained this way, however, led to a configuration with large gaps, especially around the dimer interface, that were filled with water molecules. The coordinate and topology files were therefore manually modified to remove the transmembrane water molecules and introduce additional lipid molecules to fill the gaps. We also added a 1,2-dilauroyl-*sn*-glycero-3-phosphocholine (DLPC) lipid molecule at the Tom40-Tom22 interface. Details of the final model are provided in Table S1.

### Molecular Dynamics Simulations

The simulations were performed using the CHARMM36m^55^ parameter set and explicit water was simulated using the TIP3P water model. The short-range electrostatics was calculated using a cutoff of 12 Å and the Particle-Mesh-Ewald method was used for treating long-range electrostatics.^57^ Short-range van der Waals interactions were treated using a cutoff scheme with 12 Å cutoff distance with a switching distance of 1.0 Å. All bonds were constrained using the parallel LINCS algorithm.^58^ The system was equilibrated in multiple step starting with strong restraints on the backbone (κ = 4000 kJ mol^–1^ nm^–2^), side chain (κ = 2000 kJ mol^–1^ nm^–2^) and the lipid molecules (κ = 1000 kJ mol^–1^ nm^–2^), which were gradually reduced over the course of 500 ps NVT and 5 ns NPT simulations. Finally, the fully relaxed system was simulated for an additional 2 μs for equilibration. For all simulations, we used the Nose-Hoover thermostat with a reference temperature of 300 K and a semi-isotropic Parrinello–Rahman barostat at 1 bar. The equilibration runs were performed using a 1 fs time step for the first 250 ps of NVT and 2 fs for the rest of the equilibration. For all production runs, we used hydrogen mass repartitioning to enable a 4 fs time step. The production runs were initialized from frames that were extracted from the final 500 ns of equilibration. The TOM-CC bereft of any position restraints were simulated in four independent runs of 5 μs each - referred to as the ***free*** runs in the maintext. Simulations of the stalled TOM-CC (***stalled-A*** and ***stalled-C***) were initialized from configurations extracted from the ***free*** simulations. Position restraints were applied on the backbone (κ = 100 kJ mol^–1^ nm^–2^) and side chain (κ = 25 kJ mol^–1^ nm^–2^) heavy atoms of residues 124–129 of Tom22 to model the stabilization due interactions with an underlying layer of agarose-based matrix. All simulations were performed using the GROMACS (version 2021.5) molecular simulation engine.^59^

### Applied Field Simulations

Applied electric field simulations were set up to understand the effect of the of the α2-P (open) to α2-F (constricted) transition of the α2 helix of Tom40 on the ion transport. Since only one copy of Tom40 showed the conformational transition in the α2 helix, we modeled the transition in both copies by replacing the second copy of Tom40 in the open state with the constricted Tom40 configuration. An open state of TOM-CC with both Tom40 proteins with the native α2 configuration was also simulated for comparison. Both systems were simulated using a salt concentration of 1 M KCl. Additional simulations were performed with 1 M CaCl_2_. We performed three independent applied field simulations for both the open and constricted TOM-CC system. Electric field corresponding to a potential difference of +100 mV was applied in the *z* direction that is by construction normal to the membrane plane. A positive polarity of the voltage in the present simulations indicates that the IMS side of the membrane was kept at positive voltage with respect to the cytosolic side. This specific polarity was used to model the transport of cations moving from the IMS to the cytosolic side, as was observed in the single-channel optical ion-transport experiments.^37^ The instantaneous ionic current passing through was determined using

$$I(t)=\frac{1}{l_{z}}\sum_{j=1}^{N}q_{j}(z_{j}(t+\Delta t)-z_{j}(t))$$ 1

where *z*_*j*_(*t* + Δ*t*) – *z*_*j*_(*t*) is displacement of ion *j* along the *z*-axis and *q*_*j*_ the charge on the ion. A linear fit to the plot of the cumulative current of the cationic and anionic species was used to obtain the respective currents. The net current is simply the sum of currents due to the cationic and anionic species.

### Data Analysis

Trajectory visualization and examination was done using the Visual Molecular Dynamics (VMD) software.^60^ The principal component analysis (PCA) on the backbone Cα atoms was performed using the *covar* (calculation of the covariance matrix) and *anaeig* (analysis of eigenvectors and extract projections) utilities from the GROMACS package.^59^ Additional analyses of the cross-correlation between the projections of the Tom22 helix–helix distance on the IMS (R120 Cα-Cα) and the cytosolic side (Y65 Cα-Cα) along the *xy*-plane, and the barrel ellipticity was performed using custom python scripts. For the calculation of the barrel ellipticity, the extent of the barrel along the barrel axis (reoriented with the *z*-axis) was divided into contiguous segments of 2.5 Å, and all the Cα of the barrel were allotted to each of the segments based on their *z* positions. For every frame, the spatial distribution of these atoms within each segment was projected onto the plane orthogonal to the barrel axis, and an ellipse was fitted to these 2D coordinates using a covariance analysis. The eccentricity of the ellipse - calculated as $\sqrt{1-(b^{2}/a^{2})}$ , where *a* and *b* are the semimajor and semiminor axes, respectively, was used as a quantitative measure of the local distortion. The time evolution of the eccentricity for each segment was obtained for the trajectory and used to calculate the averages and standard deviations. The analysis of the correlated fluctuations was performed using the procedure described previously using the *DyNetAn* package.^61^ Briefly, the residue–residue correlations were extracted from the input trajectory and was used to construct a network of nodes (Cα atoms) connected by edges. Two nodes are considered connected if they are within 4.5 Å of each other in at least 75% of the frames. For the network analyses, the distance measure between two connected nodes was based on the generalized correlation calculated in the first step. A dihedral PCA analysis of the ***free***, ***stalled-A*** and ***stalled-C*** runs and the pairwise Jensen-Shannon distances between backbone conformational distribution of each residue was performed using utilities provided within the *pensa* module.^62^ Pore radius was calculated using the HOLE software.^63^ The force distribution analysis was performed on the simulation trajectory using the FDA tool implemented in GROMACS.^64^ FDA was used to investigate the differences in internal forces of TOM-CC under external perturbation in the form of stabilization of the IMS end of Tom22. In FDA, the atomic pairwise forces is calculated from the simulation trajectory and the pairwise residue forces are obtained by a vector summation of the atomic forces using

$${\vec{F}}_{r_{i},r_{j}}=\sum_{i\in r_{i},j\in r_{j}}{\vec{F}}_{ij}$$ 2

where, *i* and *j* are atoms of residues *r*_*i*_ and *r*_*j*_, respectively, and *r*_*i*_ and *r*_*j*_ are distinct residues. The FDA calculations were performed on the TOM-CC ***free*** (20 μs) and ***stalled-A*** (10 μs) simulation data sets, and the forces averaged over all trajectory frames that were saved at a frequency of 80 ps. The differences between the residue pairwise forces between the two states was used to understand the force propagation under applied weak restraints on the IMS end of Tom22.

## Results and Discussion

We first conducted four independent 5 μs production molecular dynamics (MD) simulations of the *N. crassa* TOM-CC embedded in a lipid membrane to characterize the overall dynamics of the TOM-CC and its constituent subunits. The simulations were bereft of any artificial constraints on the channel or its components, thereby modeling a TOM-CC freely diffusing in the membrane.

### Tom22 Helices Exhibit Large Motion Around a Stable Hinge Region

In all our simulations, we observe significant structural deviations in both the IMS and the cytosolic helical segments of Tom22 (Figure 2A). The motion appears to be around a hinge region (P99) at a helix kink located near the center of the membrane. The cytosolic and IMS segments of Tom22 exhibit substantial flexibility with root mean squared fluctuations (RMSF) of the helix reaching up to 10 Å, whereas the transmembrane (TM) helix remains comparatively rigid (≈1 Å RMSF; Figure 2B). Despite this apparent stability, simulations reveal variability in the Tom22 TM helix-kink angle, ranging from 125 to 175°, in contrast to the 145° angle observed in the cryo-EM structure of *N. crassa* TOM complex (Figure S1). Trajectory analysis further shows that the IMS domains of the two Tom22 subunits engage in reversible salt-bridge interactions, primarily involving E116 and R120 (Figure 2A insets; Figure S2). These interactions at the IMS side likely contribute, along with thermal effects, to the observed motions of the helix. The helix flexibility is not surprising, given the poor resolution for the non-TM regions of Tom22 in the cryo-EM maps.^11,12,14^ Additionally, the variability of Tom22 at the IMS and cytosolic sides may be filtered out during image processing, eigenimage analysis, and multivariate analysis steps.

**Figure 2:**
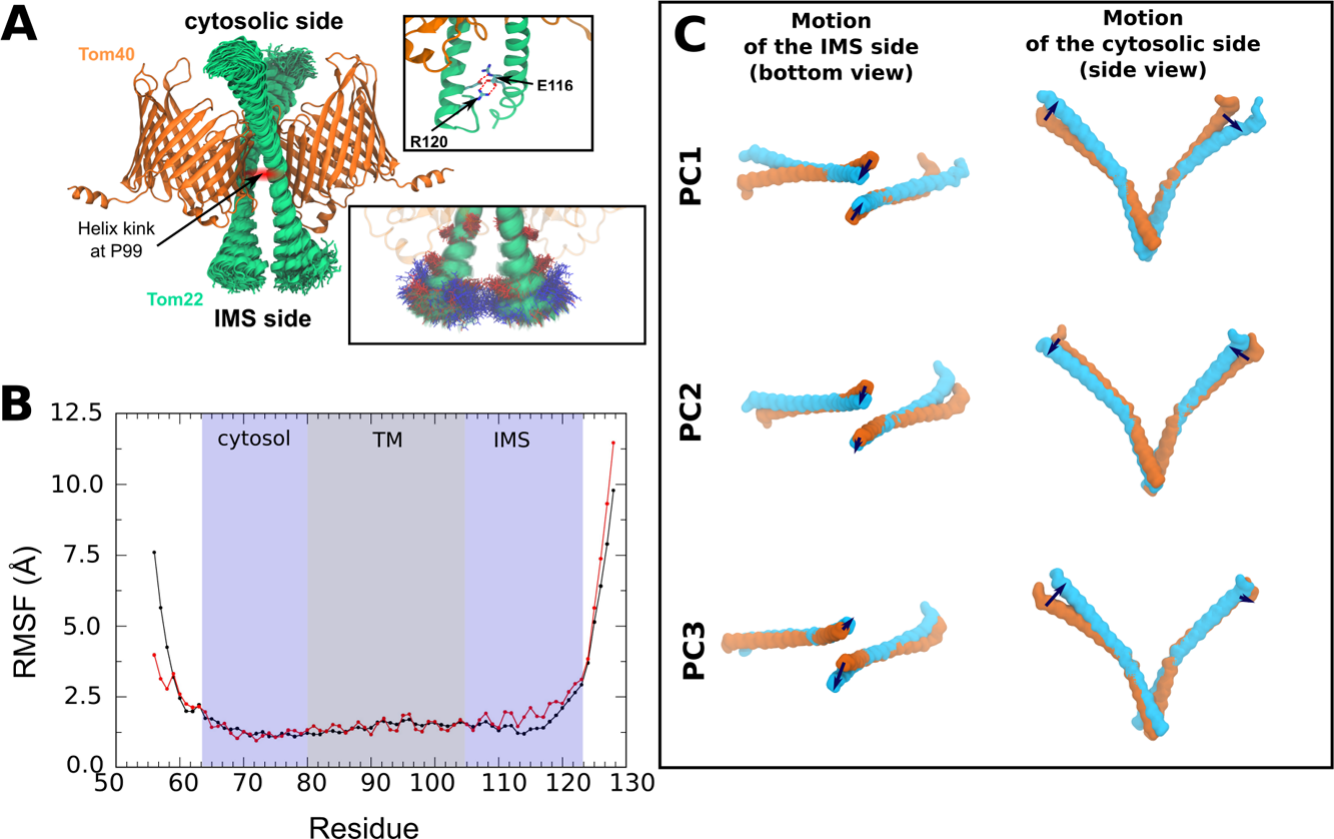
Conformational flexibility and principal motions of Tom22 helices during unbiased MD simulations. (A) Superposition of Tom22 conformations (green) extracted from unbiased simulations, highlighting dynamic variability. Insets: close-up views of the Tom22 C-terminal interactions, showing salt-bridge formation between residues E116 and R120 that stabilize the IMS ends of the helices (top) and how these interactions promote conformational variability (bottom). (B) Root-mean-square fluctuations (RMSF) of Tom22 Cα atoms, computed from trajectory data. The trajectory was aligned using least-squares fitting to the hinge region (residues 97–100) of the equilibrated starting frame. The hinge region was chosen due to its minimal motion at the interface. Red and black curves represent the RMSF for the two Tom22 monomers. Shaded regions indicate the cytosolic, transmembrane (TM), and intermembrane space (IMS) segments of Tom22. (C) PCA of Tom22 backbone dynamics. Cα atoms of residues 65–122 were used, excluding the highly flexible termini. Structures were aligned to the hinge region (residues 97–100) of the equilibrated starting frame. The dominant eigenvectors (PC1–PC3) illustrate distinct modes of motion, including concerted tilting and rocking of the helices.

Interestingly, multiple sequence alignment (MSA) of the Tom22 protein sequences suggests that the salt bridge forming residues - E116 and R120 - are present in all fungal Tom22 sequences, indicating that interaction of the IMS domains is a conserved feature (Figure S3). Animal Tom22 sequences differ in that they feature a glutamine-rich stretch, which could nonetheless mediate Tom22 interaction on the IMS side and orchestrate similar helix dynamics. Notably, on the cytosolic side, a large stretch of acidic residues is characteristic of the Tom22 subunit. This negatively charged region is largely disordered and is involved in capturing incoming proteins as well as interacting with the Tom20 receptor subunit.^24,46,52,53^ Moreover, a recent cryo-EM map of the *N. crassa* TOM complex,^14^ and more recently, of the *C. thermophilum* TOM complex,^65^ reveal distinct positions for the Tom20 receptor domain over the cytosolic mouth of the core complex, and a strong interaction between the basic patch of the Tom20 with the negatively charged cytoplasmic stretch of Tom22. The regulation of the import function is thus expected to involve a precise coordination between the inner mitochondrial membrane import machinery on the IMS side and the receptor domains on the cytosolic side.

To evaluate whether motion of the C-terminal IMS domain affects the cytosolic N-terminal region, potentially via simple rigid-body helix motion as a signal transduction mechanism,^37,40^ we computed cross-correlation coefficients between the *xy*-plane projections of two helix–helix distances between the Tom22 helices, one is the R120 Cα-Cα distance on the IMS side and the other Y65 Cα-Cα in the cytosolic side. As shown in Figure S4, the vectorial movements of the IMS and cytosolic domains are only weakly correlated across multiple lag-times considered for the analysis. However, principal component analysis (PCA) identified three dominant modes of motion (PC1, PC2 and PC3) affecting both the IMS and cytosolic regions, alongside changes in the Tom22 TM helix bend angle (Figures 2C and S4B). PC1 reflects a correlated rocking of the two Tom22 subunits; PC2 captures an out-of-plane tilt with coordinated C-terminal displacement and in-plane motion of the N-termini; PC3 reveals a subtle rigid-body-like rotation involving both termini. The observed directionality of these motions suggests a dynamic behavior that is more complex than a simple “scissor-like” motion of the Tom22 helices about the hinge.

The Tom40-Tom22 interface also accommodates a phospholipid molecule. We noted that the lipid molecule undergoes conformational fluctuations within the pocket. The lipid headgroup itself remains largely stable and forms interactions with the two lysine residues (K298) from the Tom40 barrels through the phosphate oxygens (Figures 3A and S5). However, we observed some fluctuations in the conformation localized at the glycerol backbone and the acyl chains (Figure S5). Altogether, while the lipid molecule appears to undergo minor conformational shifts within the interface pocket, we do not find evidence for large structural changes in the lipid. This is in line with the evidence from all high-resolution structures of TOM complex that contain this lipid indicating structural stability.^10–14,65^

**Figure 3:**
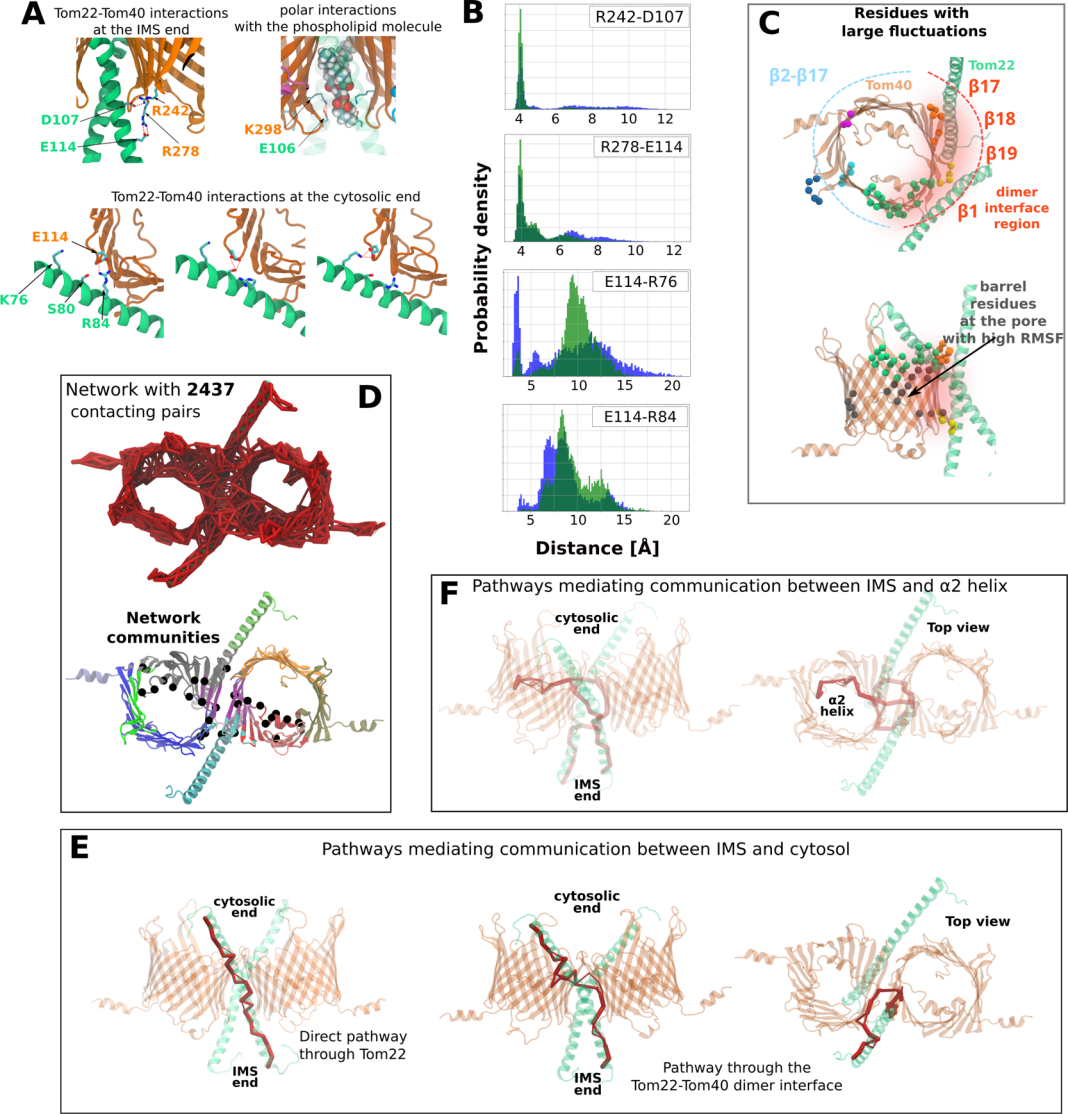
Tom40 barrel distortions are coupled to Tom22 motions. (A) Representative salt bridges formed between Tom22 (green) and Tom40 (orange) during simulations. (B) Distribution of the distances between the salt bridge forming residues between the two Tom40–Tom22 interfaces (depicted in blue and green) shows that the salt bridges undergo breakage and reformation, coinciding with conformational fluctuations of Tom22. (C) Tom40 residues exhibiting the largest Cα backbone fluctuations (RMSF > 1.25 Å), mapped onto the structure. Colored dots indicate residue clusters (Table S2). Orange and green dots mark the Tom40-Tom40 dimer interface near the cytosolic side; yellow dots mark Tom22 interactions and coordinate the interface phospholipid. (D) Dynamical network communities within the Tom40–Tom22 complex, shown in distinct colors. Black balls indicate residues involved in the 20 highest-ranked edges by betweenness centrality (Table S3). (E) Predicted communication pathways (red) connecting Tom22 IMS residues to cytosolic regions of the TOM complex. (F) Predicted signal transmission routes (red) linking the IMS end of Tom22 to the α2 helix within the Tom40 β-barrel.

### Tom40 β-Barrel Distortion Is Connected to Tom22 Helix Motion

Next, we asked whether Tom22 motions influence dynamics of the Tom40 barrels, potentially modulating their channel properties. The cryo-EM structure of the *N. crassa* TOM complex^14^ suggests that the Tom22 helix interacts with Tom40 on both the IMS and cytosolic sides via salt bridges and hydrogen bonds (Figure 3A). Our MD simulations suggest that these interactions are not static. Instead, they exist in a dynamic equilibrium, repeatedly breaking and reforming over time (Figure 3B). PCA of the Tom40-Tom22 dimer dynamics reveals dominant eigenvectors demonstrating that fluctuations of the Tom22 helices are correlated with distortions of the Tom40 β-barrels (Figure S6 and Supplementary Video). Mapping the residues showing the highest backbone fluctuations onto the Tom40 structure shows that the largest motions occur in loop regions and, unexpectedly, within β-strands β1−β5 and β16−β19 at the Tom40-Tom22 dimer interface (Figure 3C; Table S2).

Since a simple scissor-like motion of Tom22 about its hinge region was already ruled out in our analysis, we analyzed the Tom40 and Tom22 fluctuations using a generalized correlation measure, based on residue–residue mutual information as a more robust framework for studying correlated motions in protein complexes.^66,67^ The dynamic network analysis has commonly been used to identify correlated motions in protein assemblies, including mechanosensitive complexes.^68–70^ Fluctuations in Tom40 and Tom22 were analyzed and a graph based on residue–residue contacts was constructed with the connected edges weighted using the mutual information measure.^61^ The resultant network consisting of 2,437 contacting residues and edge weights revealed distinct communities, each consisting of nodes that are considered well-connected within the network (Figure 3D). Notably, strands β1, β17, β18, and β19 from both Tom40 subunits form a single, cohesive community (colored magenta) within the complex. Fluctuations of helix α2 show strong correlation with those of the adjacent β-strands β10−β15, consistent with cryo-EM data indicating interactions between these regions.^10–14^ To further evaluate the functional significance of residues at the Tom40-Tom40 interface and the role of Tom22 as a potential transceiver module, we computed the betweenness centrality of the network edges, a metric that identifies key residues (nodes) and interactions (edges) involved in propagating conformational changes. Nodes linked to the highest centrality values were predominantly located within the α2 helix and the Tom40–Tom40 interface (black beads in Figure 3D; Table S3), indicating that these regions form a communication hub responsive to Tom22-mediated conformational dynamics. Using R120 and Y65, located at the IMS and cytosolic termini of the two Tom22 helices, respectively, as source and target nodes, the optimal communication pathway for transmitting conformational changes was found to run directly along the Tom22 helix (Figure 3E, top panel). Notably, alternative lower-ranked paths traverse the Tom40-Tom22 dimer interface that incorporate residues from both the subunits (Figure 3E, bottom panels; Table S4). These findings suggest that Tom22 enables both direct and indirect routes (through Tom40) for communication within the TOM-CC.

Interestingly, our analysis highlights the α2 helix as a strongly connected component within the dynamical network, implying that the helix structural state and fluctuations may be responsive to the structural state (Figure 3D). The intimate connection of the α2 helix with the overall complex dynamics emerging from our network analysis can also be observed as a communication pathway shown in Figure 3F. The exact residues that are part of these pathways are specified in Table S4. This emergent feature from the network analysis is particularly intriguing, given the crucial role of the N-terminal segment within Tom40 in the protein import function. Note that the pathways calculated this way are the ones with the greatest weights and represent a direct connection. Although the α2 helix does not appear to participate in the direct pathways involved in communication between the IMS and cytosolic ends of Tom22 helices, it is likely sensitive to the overall dynamics of the complex. The N-terminal segment consists of the α1 helix at the IMS exterior and α2 that is stabilized at the center of the barrel, the latter contributing to a narrow pore lumen (Figure 4A). Estimates of the Cα backbone fluctuations of 1–2 Å from our simulations (Figure 4B) reflect strong hydrophobic and polar interactions anchoring α2 to the inner wall of the Tom40 β-barrel. In contrast, helices α1 and α3, positioned outside the barrel, show substantially greater mobility. This is consistent with their high sequence variability across species (Figure S7) and their observed absence from the cryo-EM density maps of Tom40.^11–14^

**Figure 4:**
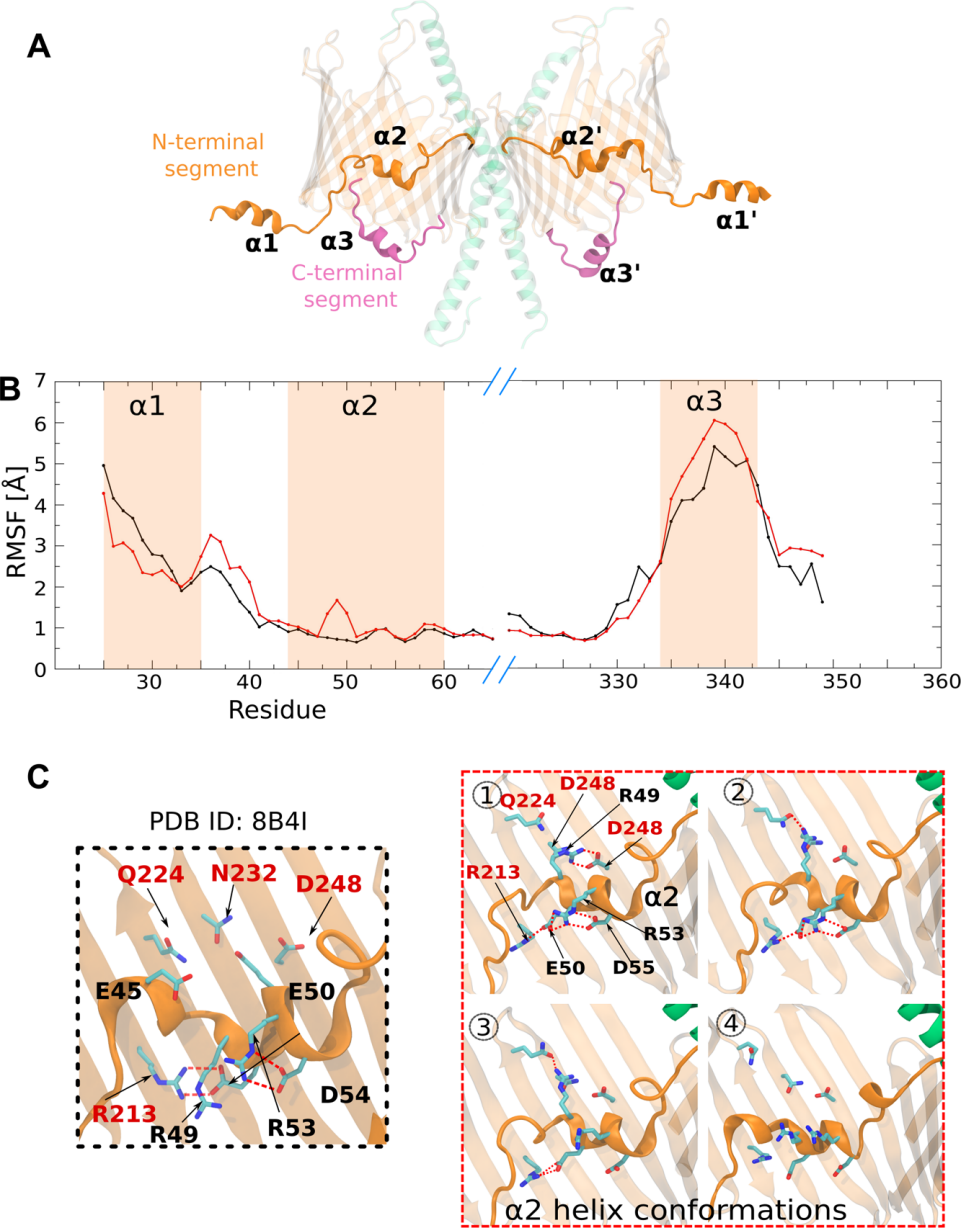
Structural and dynamic configurations of helix α2 within the Tom40 β-barrel. (A) N and C-terminal segments of Tom40 containing the helical regions α1, α2, and α3. (B) Root-mean-square fluctuations (RMSF) of Cα atoms in helices α1, α2, and α3 of Tom40, highlighting the relative rigidity of α2. (C) Left panel shows residues of the α2 helix and its interactions with residues of the barrel wall (labeled in red) within the Tom40 β-barrel in the cryo-EM structure (PDB ID: 8B4I). The panels 1–4 on the right show the side-chain conformations of key α2 residues and their interaction partners sampled during simulations. All residues are depicted as sticks with interactions shown as red dotted lines.

Surprisingly, despite the low Cα backbone fluctuations of α2, we observed notable torsional flexibility in residues 48–50, accompanied by distinct hydrogen-bonding patterns between α2 and adjacent β-strands (Figure 4C). We note the significant differences in side-chain conformations of residues R49 and E50 between the two monomers, which affect the backbone fluctuations. This may be attributed to the inconsistent sampling of these conformational states between the monomers. It appears that the observed network of interactions directly influences the fluctuations in the α2 helix. Notably, the interacting residues (E45, R49, R53, D54, R213, and D248) are conserved across fungal and animal Tom40 homologues (Figure S7), suggesting also that their alternative conformations may play a functional role.

### Stabilization of Tom22 Unlocks Alternative TOM-CC States

The simulations in the previous sections (hereafter referred as free) are akin to the freely diffusive state of TOM-CC in the experimental TIRF setup used for the tracking of TOM-CC and its ion-transport behavior.^37^ Intriguingly in these experiments, it was shown that the intermittent stalling of the TOM-CC at arbitrary positions due to interactions with an immobilizing phase below the membrane is correlated with the transition from a state of high Ca^2+^ ion flux to states with reduced flux (Figure 1B). Therefore, we next examined how restricting the movement of the IMS domains of Tom22 affects TOM-CC dynamics within the Tom40 barrel lumen. To this end, we performed simulations of TOM-CC with restraints on the IMS end of Tom22 (hereafter referred as ***stalled***) to investigate the differences in the dynamics of the complex in the ***free*** and ***stalled*** states. In the ***free*** TOM-CC runs, we observed that the IMS ends of Tom22 undergo large fluctuations that appear to be mechanistically connected to the Tom40 dynamics. However, it is not clear which of the states of Tom22 would correspond to the stalled configuration stabilized by inner membrane components. We therefore selected two representative configurations from the ***free*** simulations: one with the IMS ends of Tom22 placed **a**part and noninteracting (***stalled-A***), and another with them **c**losely associated (***stalled-C***) (Figure 5A). Simulations were performed with weak restraints on the C-terminal residues (124–129) of Tom22. Restraints on the Tom22 induced significant conformational changes in the Tom40 β-barrel over 10 μs, reflected in altered barrel ellipticity, particularly toward the IMS end of the barrel (Figure 5B). The ellipticity plots also show that under the stabilization effects via Tom22, the central and IMS regions of the barrel undergo larger deformations compared to the cytosolic side. Thus, the IMS side of the barrel appears to be more responsive to the (de)­stabilization state of the Tom22 helices. Dihedral PCA highlighted distinct dynamic shifts in both Tom22 and Tom40 for the ***stalled*** simulations relative to the unrestrained ***free*** state. Simulations of the Tom40–Tom22 complex combined with PCA confirmed that the ***stalled-A*** and ***stalled-C*** systems sample more restricted configurational space compared to the ***free*** simulations (Figure S8, left panels). Moreover, the PC1-PC2 distribution shows that ***stalled-A*** and ***stalled-C*** systems sample distinct configurations. The same conclusions are obtained with dihedral PCA performed only on the intrapore N-terminal helical segment of Tom40 (Figure S8, right panels). Interestingly, both the PCA data sets indicate that the ***stalled-A*** simulation can access additional configurational space not sampled in either the ***free*** and ***stalled-C*** simulations of TOM-CC.

**Figure 5:**
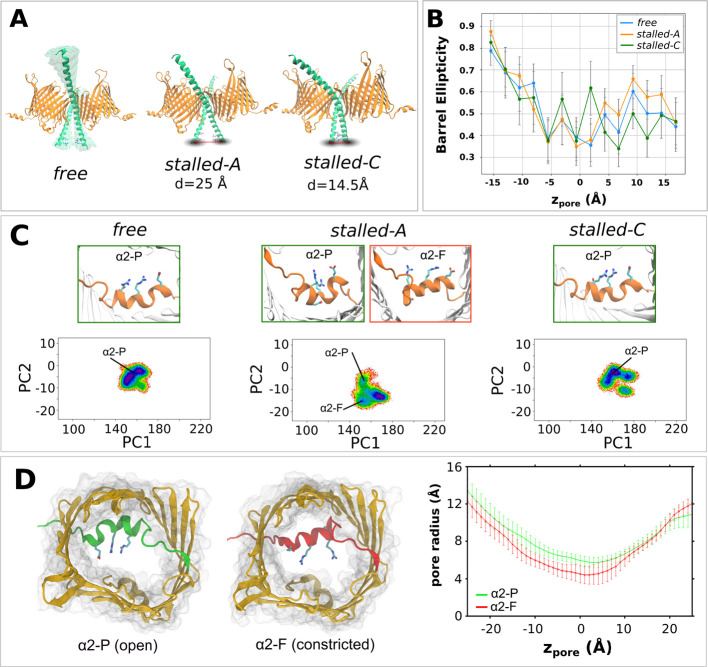
Conformational dynamics of the TOM-CC complex under Tom22 C-terminal restraints. (A) Schematic overview of the three simulated configurations: ***free*** (unrestrained), ***stalled-A*** (noninteracting Tom22 IMS ends), and ***stalled-C*** (interacting IMS ends). (B) Ellipticity profiles of the Tom40 β-barrel along the pore axis in the three simulation conditions. (C) Representative conformations of the α2 helix (top) and projection of backbone torsional dynamics of the α2 region onto the first two principal components (PC1 and PC2) from dihedral PCA. A transition from a bent partial helix (α2-P) to a full-length conformation (α2-F) is observed exclusively in ***stalled-A***. (D) Structural models of Tom40 in α2-P and α2-F conformations (left) and corresponding pore radius profiles computed with HOLE (right).

Visual inspection of the trajectories reveals a prominent structural transition in the α2 helix backbone in case of ***stalled-A*** simulations runs, which was not observed in the ***free*** and ***stalled-C*** runs (Figure 5C). Specifically, a transition of α2 from a bent partial helix (α2-P) configuration to a full helix conformation (α2-F) is observed in one of the copies of the Tom40 subunit. PCA analysis using the backbone dihedral of the N-terminal region and the Cα distances of the α2 helix to the opposite wall of the β-barrel also shows that in the ***stalled-A*** simulations the α2 helix samples distinct conformational state not observed in the ***free*** and ***stalled-C*** runs (Figure 5C). This transition disrupts α2-barrel wall interactions, including the E50-R213 salt bridge, and involves torsional shifts in T46, A48, R49, and V51 (Figure S9), coinciding with a pore radius reduction from 5.7 to 4.4 Å at its narrowest point (Figure 5D).

To quantify backbone torsion differences in α2, we calculated Jensen-Shannon distances between the backbone torsional distributions observed in the simulations.^62^ The greatest variations between ***free***, ***stalled-A***, and ***stalled-C*** states localized to Tom40 β-strands interacting with Tom22 and helical segments including α2 (Figure S10, Table S5). Moreover, residue–residue correlation and network analyses of the ***stalled-A*** configuration revealed that 14 of the 20 highest betweenness centrality edges were conserved from those observed for the ***free*** state (Table S6), indicating robust and common information flow pathways for all the TOM-CC states. Analysis of the conformational states of these residues from the simulations in different conditions revealed differences in their dynamics and interactions (Figure S11). Particularly, ***stalled-A*** exhibited a loss of R213 interactions with the P41 backbone and E50 side chain that stabilize α2 in the ***free*** and ***stalled-C*** states (insets in Figure S11). Additionally, the list of edges with highest betweenness centrality features a common F309-M324 interaction, in contact with the central phospholipid, that links the Tom40 dimers and likely facilitates interdomain communication (Figure S12). While conformational fluctuations are observed in the central phospholipid (Figure S5), it is not clear if it plays an active role in the TOM complex dynamics. In general, phosphatidylcholine, which is a major bilayer-forming component of the OMM, has been reported to be critical for the TOM complex biogenesis.^71^ We additionally calculated the inter-residue forces in the TOM-CC upon the application of the stalling restraints.^72^ This revealed a set of residue interactions showing the greatest changes in the force between the ***free*** and the ***stalled-A*** state (Figure S13A). The largest force differences (250 pN cutoff) within the network were observed for residues in the α2 helix and barrel residues on the opposite barrel wall (β2−β5). The residues involved in these interactions are depicted as yellow beads in Figure S13B. These residues appear to form a connection between the α2 helix and the Tom22-Tom40 interaction site (red beads). On decreasing the cutoff to 75 pN, we see that the force signal spreads out to the Tom22 IMS domain (Figure S13C). Considering that the simulations apply the perturbation to the IMS domain of the channel, it appears that the signal originates from small force differences at the IMS end of Tom22 that gradually amplifies around the β2−β5 wall and the α2 helix as it propagates through the structure. It must be noted here that the pathways constructed using force differences reveal how the force redistributes during mechanical stress transfer. This, together with the results from the correlation network analysis, suggests that the Tom22 and Tom40 subunits could be coupled both dynamically and mechanically.

Overall, the examination of the ***free*** and ***stalled*** simulations suggest a mechanism whereby the stabilization of Tom22 in the IMS side influences the dynamics of the TOM-CC, prominently of the Tom40 pore subunit. The stabilization of the Tom22 appears to bias the transition of the pore subunit toward alternate conformational states with a significantly narrow pore radius. This suggests a mechanosensitive behavior of the TOM complex, whose behavior could be modulated through interactions with other biomolecular actors on the IMS side that partake in the mitochondrial import pathway.

### Conformational Changes in the α2 Modulate Tom40 Ion Conductance

As the final step, we sought to functionally link the Tom22-dependent structural changes observed in the Tom40 intrapore helix α2 to ion permeation through the TOM channel. Consistent with our goal to unravel the mechanistic basis of Tom22-Tom40 interactions and their role in conformational regulation of the complex, we employed computational electrophysiology on the two TOM-CC states representing distinct α2 conformations: the open (α2-P) and constricted (α2-F) forms (Figure 5D). While the applied field simulations do not replicate the electrochemical environment of the outer mitochondrial membrane, they allow for direct quantification of ion flux. To this end, we constructed two TOM-CC configurations, one with both the Tom40 subunits in the open state and the other with both of them in the constricted state as described in Methods. Following the polarity of optical ion flux assays (Figure 1B), we applied a potential of +100 mV across the TOM-CC with the IMS side held positive. Simulations were performed in the presence of 1 M KCl and 1 M CaCl_2_. The cumulative current over simulation time plotted in Figure 6A shows a reduced cation flow in case of the constricted state of Tom40. We estimated a potassium current of 0.33 ± 0.01 nA for the open and 0.15 ± 0.02 nA for the constricted state - a reduction of the current by half. A similar trend was also observed for the calcium current with values of 0.15 ± 0.01 and 0.08 ± 0.01 nA for the open and constricted state, respectively. The chloride current however does not change between the open and constricted states with a current of 0.15 ± 0.1 nA with 1 M KCl and 0.25 ± 0.01 nA with 1 M CaCl_2_. The reduction in potassium and calcium ion current suggests that the alternative configuration of the α2 helix observed in these simulations is likely one of the possible states with reduced cation flux. However, we only observe a partial drop (∼50%) in the cation flux, whereas the optical ion flux assay^37^ also reported a low conducting state with a low residual Ca^2+^ flux. It is possible that additional conformational states of α2 corresponding to the fully closed state were not sampled in our simulations and would require advanced biased simulation techniques. Nonetheless, the results provide a structural basis for the experimentally observed reduction in Ca^2+^ flux in the case of stalled TOM-CC. Analysis of the cation density within the channel for the open and constricted configurations revealed that the conformational transition in the α2 helix brings about a change in electrostatics at the Tom40 pore that ultimately changes the cation flow (Figure 6B). In the case of the open conformation, the cation flux is mediated via two pathways. The first involves the anionic residues on the helix face of the barrel (E45 and E147), and the second employs interaction with residues on the barrel wall (D68, E329, and E334) opposite to the helix face. The α2-F to α2-P transition leads to a shift in the side-chain orientation of the D45 residue away from the pore lumen. This retracted conformation of D45 is stabilized by hydrogen bond interaction with the residue Q192 (Figures 6C and S14). The conformational change leads to a shift in the pore electrostatics that ultimately affects the cationic flux.

**Figure 6:**
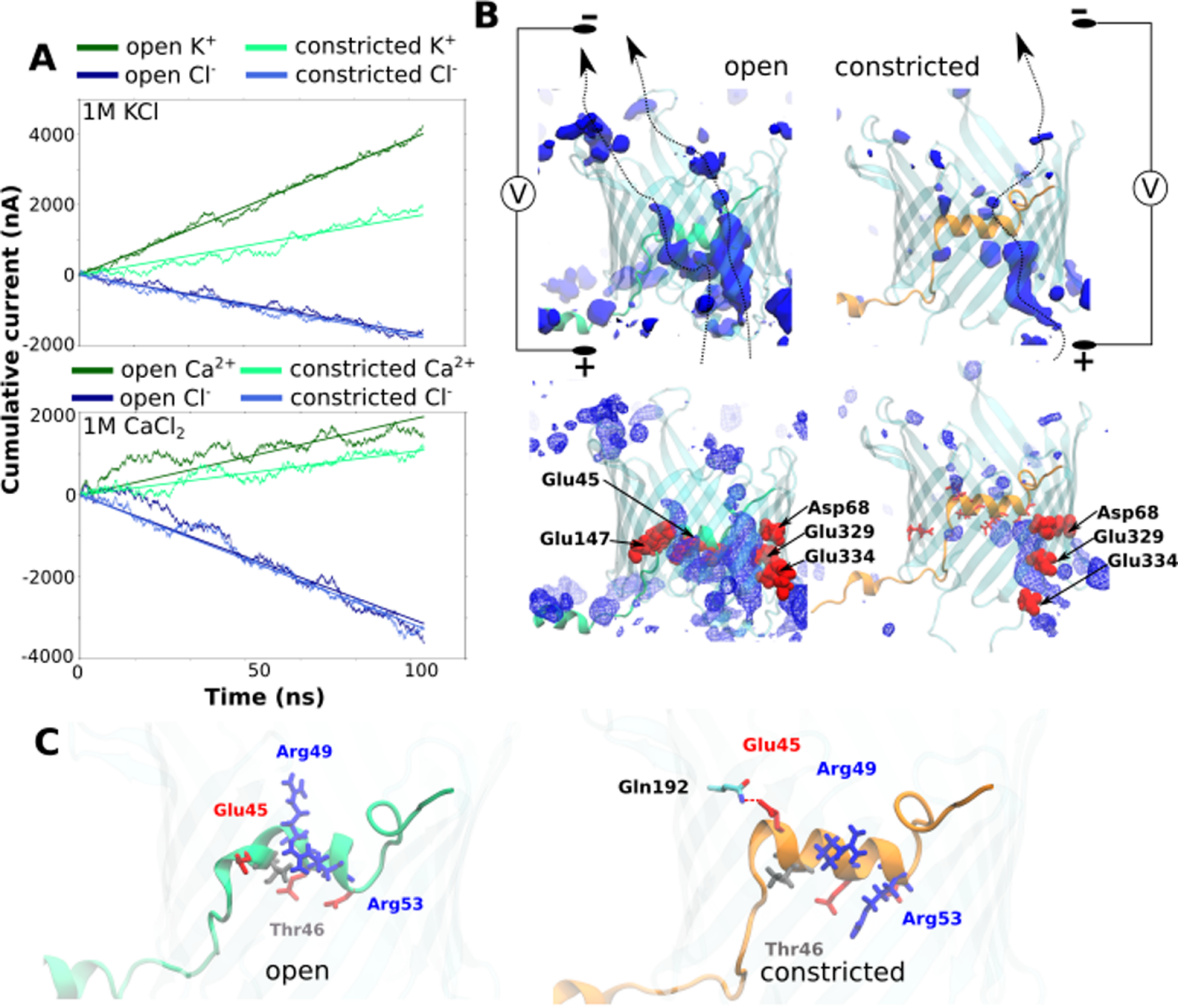
Ion-transport properties of the TOM-CC from applied field simulations. (A) Simulated cation (K^+^, Ca^2+^) and anion (Cl^–^) currents in the TOM-CC complex adopting the α2-P (open) and α2-F (constricted) conformations, under 1 M KCl or 1 M CaCl_2_ electrolyte conditions. (B) Cation density maps (blue) within the Tom40 pore, highlighting preferred ion translocation pathways (arrows). Red space-filling spheres denote anionic residues contributing to cation coordination. (C) Representative side-chain conformations of the key residues of the α2 helix in the α2-P and α2-F states, illustrating conformational rearrangements associated with altered pore conductance.

Together, these results establish a direct link between Tom22-dependent conformational transitions of the α2 helix and the modulation of ion conductance through Tom40. This suggests that Tom22 functions not only as a structural adaptor at the Tom40-Tom40 interface but also as a dynamic regulator of pore activity within the TOM complex.

## Conclusions

Tom22 has long been recognized as a central component of the TOM complex, serving as a receptor domain, a docking site for Tom20, and a key structural element of the TOM-CC dimer.^14,24,46,52,53^ The primary aim of this study was to elucidate the molecular basis of mechanosensitive behavior of TOM, as suggested by recent optical ion flux recordings of TOM-CC embedded in planar lipid membranes, comparing restricted and freely diffusing states.^37^

Our simulations reveal that the IMS domains of Tom22 act as dynamic sensing elements, closely coupled to conformational changes in the intrapore α2 helix of Tom40. This establishes a mechanistic link between Tom22 flexibility, α2 helix transitions, and modulation of ion conductance. Tom22 thus functions not only as a structural scaffold but also as a dynamic regulator of TOM activity. Notably, simulations with restrained Tom22 motion (***stalled-A***) show that immobilization of Tom22 drives structural transition in the intrapore α2 helix of Tom40 leading to a more constricted pore, associated with reduced cation flux. This is consistent with experimental observations of decreased Ca^2+^ conductance.^37^ These results suggest that Tom22 can act as a mechanical effector, modulating the structural and conductive states of the pore. Given the associated changes in pore geometry and electrostatics, this mechanism is likely to influence precursor translocation efficiency.

Previous studies have shown that the Tom20 receptor subunit interacts directly with the cytosolic domain of Tom22.^14,24,25,65^ Our simulations reveal large-scale fluctuations in this domain of Tom22 that could impact Tom20 positioning. Supporting this, cryo-EM maps have identified Tom20 in multiple conformations above the pore^14,65^ and cross-linking data indicate direct Tom20–Tom40 interactions.^26^ Together, these observations suggest that presequence transfer may be regulated through coordinated dynamics involving Tom22.

Tom22 is also known to associate with TIM subunits to form a TOM-TIM23 supercomplex.^5,73–75^ While such coupling is widely considered essential for precursor handover, direct evidence for regulatory feedback, such as structural gating of TOM by inner membrane components, remains limited. Our findings suggest that the transmembrane segments of Tom22 serve as a central communication hub within the TOM complex, capable of transmitting mechanical cues from the IMS to the cytosolic side. This raises the possibility that regulatory signals from the inner membrane might influence even the earliest stages of import, including presequence handover from cytosolic receptors to Tom40. Given that the ***free*** TOM complex interacts with TIM23 via the IMS domain of Tom22 during precursor translocation, the ***stalled*** TOM state is associated with distinct dynamics in Tom40, resulting in changes to pore geometry and electrostatics. This ***stalled*** state may help prevent presequence backsliding while the TOM complex awaits engagement with TIM23 for downstream translocation. From our simulations, the radius of the constricted state of Tom40 is 4.4 Å which suggests that linear peptides can fit into the Tom40 lumen. Linear peptides have been found to be accommodated in narrower channels like OmpF (3.4 Å).^76^ This is also suggested through our models of a precursor peptide sequence within the constricted Tom40 (Figure S15).

Together, these findings provide an initial understanding of the dynamics underlying the TOM complex regulation and offer new perspectives on how Tom22 might coordinate mechanistically the critical early steps of mitochondrial protein import. The study yet again demonstrates the utility of MD techniques in addressing biological questions and uncovering the molecular mechanisms underlying macroscopic properties of complex biomolecular systems. Starting from TOM-CC conformation from the cryo-EM structure which represents the open state, we could recover distinct states including one with a low cationic flux. Aside from corroborating experimental results on the mechanosensitive nature of TOM, these simulations also reveal aspects of TOM dynamics that can be formulated as testable hypothesis for further experimental investigations. The present study however has some limitations. Foremost, the Tom20 (and Tom70) receptor subunits were excluded from our simulation models. It is possible that the inclusion of Tom20 into our model could lead to somewhat altered dynamics of the cytosolic domain of Tom22. However, the important conclusions on the Tom22 flexibility on the cytosolic end are not expected to change substantially, particularly given the strong support for this in recent structural investigations.^14,65^ It would certainly be interesting to study the full TOM complex in future studies to understand the dynamics of the complete complex and model the presequence translocation process. Moreover, considering that we did not observe a full closure of the Tom40, as indicated by a 50% decrease in the cation flux, it is possible that the alternate conformation of the α2 helix (α2-F) is only one of the intermediate states. More advanced simulation approaches would be needed to boost the sampling of the alternative states. The role of lipids in the overall dynamics would also be interesting. Recent work reports a significant enrichment of cardiolipin around the complex in simulations.^77^ Furthermore, lateral pressure along the membrane has also been suggested to provide external force driving the complex dynamics and aid the transport process.^40^ Studies on the TOM in a more complex lipid environment mimicking the OMM could shed light on the role of lipid. Nonetheless, we expect that the novel insight from this study provides a useful benchmark for future simulation studies. While we have discussed the implications of our results on the protein import process and its regulation, it would be exciting to combine enhanced sampling schemes with possibly coarse-grained approaches to study the presequence transport through TOM.

## Supplementary Material





## Data Availability

Softwares used in this work are available free of charge; GROMACS (https://www.gromacs.org/); PLUMED (https://www.plumed.org/); Dynetan (https://github.com/melomcr/dynetan); HOLE (https://www.holeprogram.org/), and FDA (https://github.com/HITS-MBM/gromacs-fda/tree/release-2021-fda). Visual Molecular Dynamics program used to conduct the structural analyses of trajectory models is also available freely at https://www.ks.uiuc.edu/Research/vmd/. Plots were generated using the Xmgrace software available at https://plasma-gate.weizmann.ac.il/Grace/. Additional data for the unbiased, restrained, and applied field simulations are publically available on the Zenodo server (https://zenodo.org/records/16629051).

## Notes

Data from the analyses of the free and stalled MD trajectories and the applied field simulations (PDF)
Trajectory for the Tom22/Tom40 structural transition along the top three eigenvectors obtained from the principal component analysis (MP4)
