# Supplementary material for: Dynamic Coupling between Tom22 Motions and Tom40 Pore Dynamics Modulates Ion Transport in the Mitochondrial TOM Complex
Source: J Chem Inf Model. 2025 Oct 31;65(22):12475–88. doi: 10.1021/acs.jcim.5c01761 (PMC12648644; doi:10.1021/acs.jcim.5c01761)
Supplement: Supplementary file 1 [file ci5c01761_si_001.pdf]

Supporting Information: Dynamic coupling between Tom22 motions and Tom40 pore dynamics modulates ion transport in the mitochondrial TOM complex

Abhishek Acharya,*,† Stephan Nussberger,‡ Shuo Wang,‡,¶ and Ulrich Kleinekathöfer*,†

†*School of Sciences, Constructor University, Campus Ring 1, 28759 Bremen, Germany*

‡*Department of Biophysics, Institute of Biomaterials and Biomolecular Systems, University of Stuttgart, Stuttgart, Germany*

¶*Department of Bionanoscience, Kavli Institute of Nanoscience Delft, Delft University of Technology, Delft, The Netherlands*

E-mail: aacharya@constructor.university; ukleinekathoefer@constructor.university

Table S1: Details of the simulation system used in this study.

Simulation	Modeled Chains	Lipid	Water	Ions	System Size	Natoms
Unbiased MD	Tom40 (25-349), Tom22 (56-129),<br>Tom5 (5-50), Tom6 (5-60), Tom7	DPhPC 839<br>DLPC 1	156400	K^+ 197<br>Cl^- 201	19.5x19.5x15.8	616217
Applied-Field<br>(1M KCl)	Tom40 (25-349), Tom22 (56-129),<br>Tom5 (5-50), Tom6 (5-60), Tom7	DPhPC 839<br>DLPC 1	153900	K^+ 1297<br>Cl^- 1301	19.4x19.4x15.8	610623
Applied-Field<br>(1M CaCl2)	Tom40 (25-349), Tom22 (56-129),<br>Tom5 (5-50), Tom6 (5-60), Tom7	DPhPC 839<br>DLPC 1	152587	Ca^{2+} 1296<br>Cl^- 2596	19.4x19.4x15.8	608035

Table S2: Residues of Tom40 β-barrel showing the greatest RMSF values during unbiased simulations of the TOM-CC. The color codes correspond to different clusters of residues identified on the barrel as depicted in Figure 3C.

Color	Residues
	K298,G301,A302,A303,P304
	M87,G88,E89,R90,L91,N92,P93,L111,D112,N113,E114,G115,G138,G139,G140,Q141,D142
	D314,H315,V316,T317,Q318,Q319,A320,K321
	F166,L167,D168,G169,G170
	Q226,A227,Q228
	W125,G126,D127,R128
	K71,F85,A86,Y94,A95,F96,A97,A98,G109,N110,V305,E329,I130,T131,E147,H148,E149

Table S3: Top 20 network edges with the highest betweenness centrality obtained from the network analysis of the simulation of TOM-CC in the *free* state. Node 1 and Node 2 are the residue nodes connecting the edge. The subunit name is specified within the brackets. In the case of the edge connecting M324(Tom40) and F309(Tom40'), the apostrophe specifies the second copy of Tom40.

Node 1	Node 2
Y60 (Tom40)	F62 (Tom40)
I47 (Tom40)	R49 (Tom40)
T46 (Tom40)	R53 (Tom40)
R49 (Tom40)	R53 (Tom40)
I44 (Tom40)	T46 (Tom40)
R283 (Tom40)	C294 (Tom40)
R53 (Tom40)	Y60 (Tom40)
R53 (Tom40)	R283 (Tom40)
V69 (Tom40)	L94 (Tom22)
R49 (Tom40)	D54 (Tom40)
F62 (Tom40)	M324 (Tom40)
M324 (Tom40)	F309 (Tom40')
R66 (Tom40)	F85 (Tom40)
L56 (Tom40)	D248 (Tom40)
E50 (Tom40)	R213 (Tom40)
I328 (Tom40)	L94 (Tom22)
T70 (Tom40)	I328 (Tom40)
T46 (Tom40)	R49 (Tom40)
I44 (Tom40)	S158 (Tom40)
L56 (Tom40)	Y60 (Tom40)

Table S4: Optimal paths connecting the specified nodes of interest were calculated from the full network using the Floyd–Warshall algorithm. The number within the bracket specifies the subunit; 22 for Tom22 and 40 for Tom40, and the apostrophe indicates the second copy of the subunit.

Predicted communication pathways	Residue
R120 (Tom22) to Y65 (Tom22)	R120(22), R118(22), E114(22), A111(22), Q108(22), E106(22), K298 (40'), L296(40'), F309(40'), M324 (40), G64(40), R66(40), F85(40), Y94(40), G83(22), F81(22), L79(22), K76(22), T72(22), S69(22), Y65(22)
R120 (Tom22) to E45 (Tom40 α2)	R120(22), E116(22), M113(22), A111(22), D107(22), E106(22), A302(40'), L300(40'), K298(40'), L296(40'), T308(40'), A310(40'), L322(40'), G64(40), F62(40), Y60(40), L56(40), D54(40), Q52(40), R49(40), I47(40), T43(40), E45(40)
R120 (Tom22') to E45 (Tom40 α2)	R120(22'), R118(22'), E114(22'), A111(22'), N109(22'), E106(22'), I102(22'), F100(22'), V98(22'), L94(22'), F85(40), R66(40), F62(40), Y60(40), L56(40), D54(40), Q52(40), R49(40), I47(40), T43(40), E45(40)

Table S5: Residues of Tom40 with the highest Jensen-Shannon distance (JSD) between their backbone torsional distribution extracted for the given pair of ensembles.

Ensemble Pair	Residues with JSD $>$ 0.4
free/stalled-A	F26, G37, T46, A48, N92, D112, N113, G115, L117, G126, F135, F166-G170, D241, D334, L335, Q338, G341, P348
free/stalled-C	F26, F36, G37, T46, A48, R49, R53, E89, N113, E114, F166, G169, G170, M260, G261, Q338-G341, S344,
stalled-A/stalled-C	A25, F26, E33, Q35, S39, N40, T43, T46, A48, R49, V51, R53, K71, M87, G88 E89, N92, G115, A116, L117, G126, L167, D168, D241, R242, S258, M259, M260, G261

Table S6: Top 20 network edges with the highest betweenness centrality obtained from the network analysis of the simulation of TOM-CC in the *stalled-A* state. Node 1 and Node 2 are the residue nodes connecting the edge. The subunit name is specified within the brackets.

Node 1	Node 2
**Y60** (Tom40)	**F62** (Tom40)
**R49** (Tom40)	**R53** (Tom40)
**R53** (Tom40)	**Y60** (Tom40)
**T46** (Tom40)	**R53** (Tom40)
**I47** (Tom40)	**R49** (Tom40)
**I44** (Tom40)	**T46** (Tom40)
**F309** (Tom40)	**M324** (Tom40')
**F62** (Tom40)	**M324** (Tom40)
**T70** (Tom40)	**I328** (Tom40)
**R53** (Tom40)	**R283** (Tom40)
T70 (Tom40)	Q80 (Tom40)
I328 (Tom40)	V98 (Tom22)
**E50** (Tom40)	**R213** (Tom40)
**R283** (Tom40)	**C294** (Tom40)
**L56** (Tom40)	**D248** (Tom40)
D112 (Tom40)	A116 (Tom40)
Y94 (Tom40)	D112 (Tom40)
G83 (Tom40)	W87 (Tom40)
**V69** (Tom40)	**L94** (Tom22')

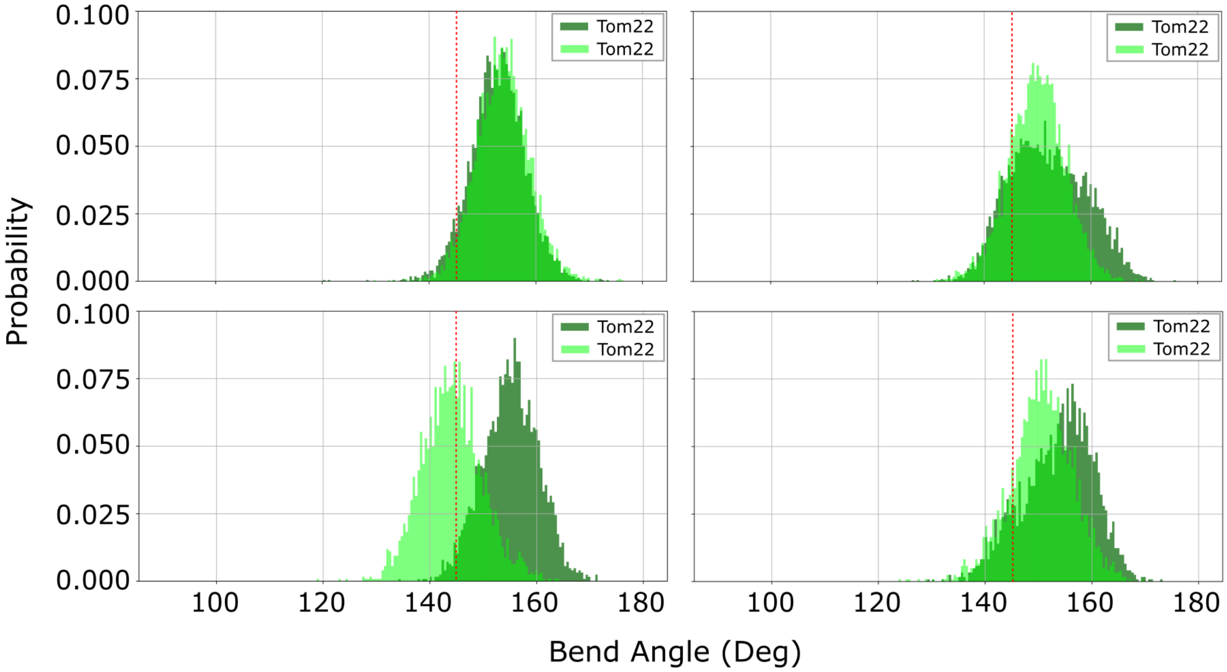

Figure S1: The distribution of the bend angle of the Tom22 and Tom22' helices from independent unbiased simulations. We report here values calculated for the angle Cα67-Cα99-Cα124, where the subscript is the residue number. The red dotted line denotes the bend angle of 145° measured from the cryo-EM structure.

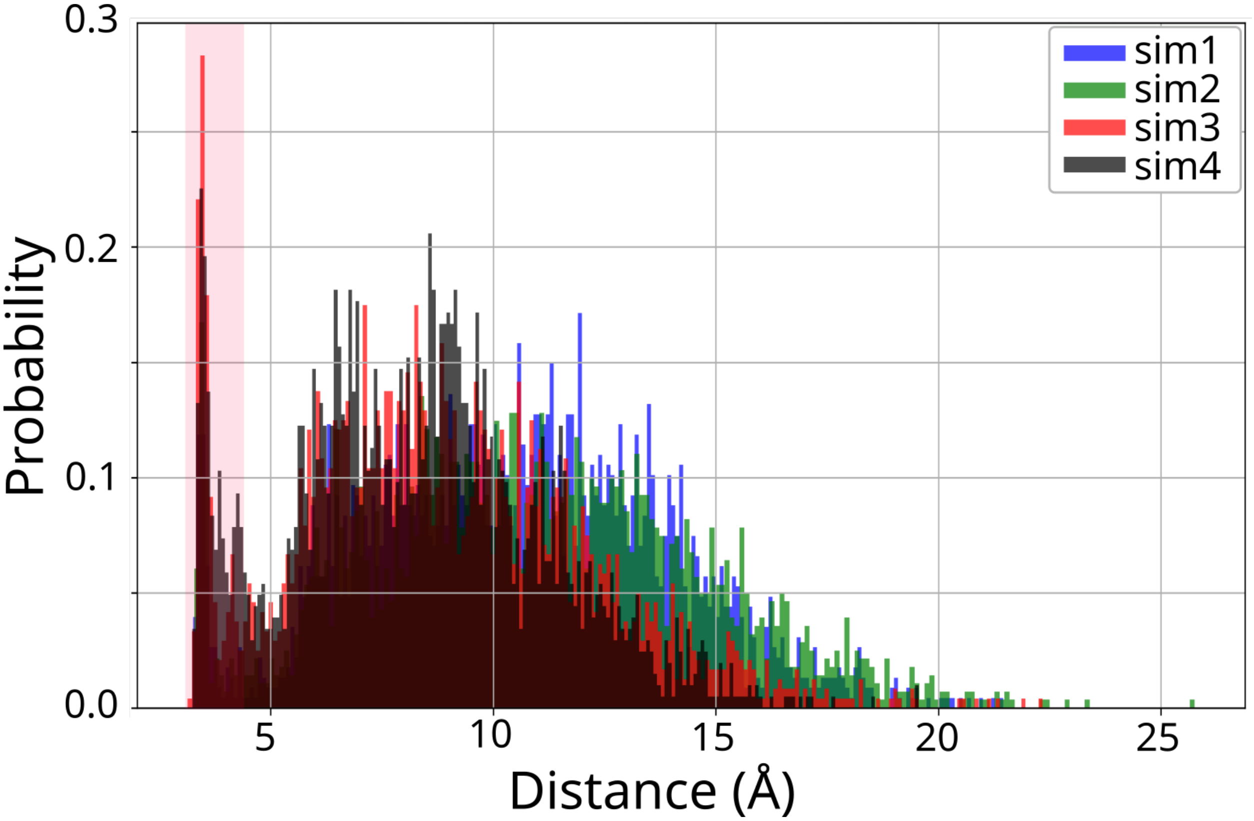

Figure S2: The distributions of the distance between E116 and R120 residues that form a salt-bridge interaction on the IMS side of the Tom22 helix. The complex is observed in two possible states where the helices form a salt bridge (distance=3.0 Å) or are in a wider distribution of non-interacting Tom22 helices.

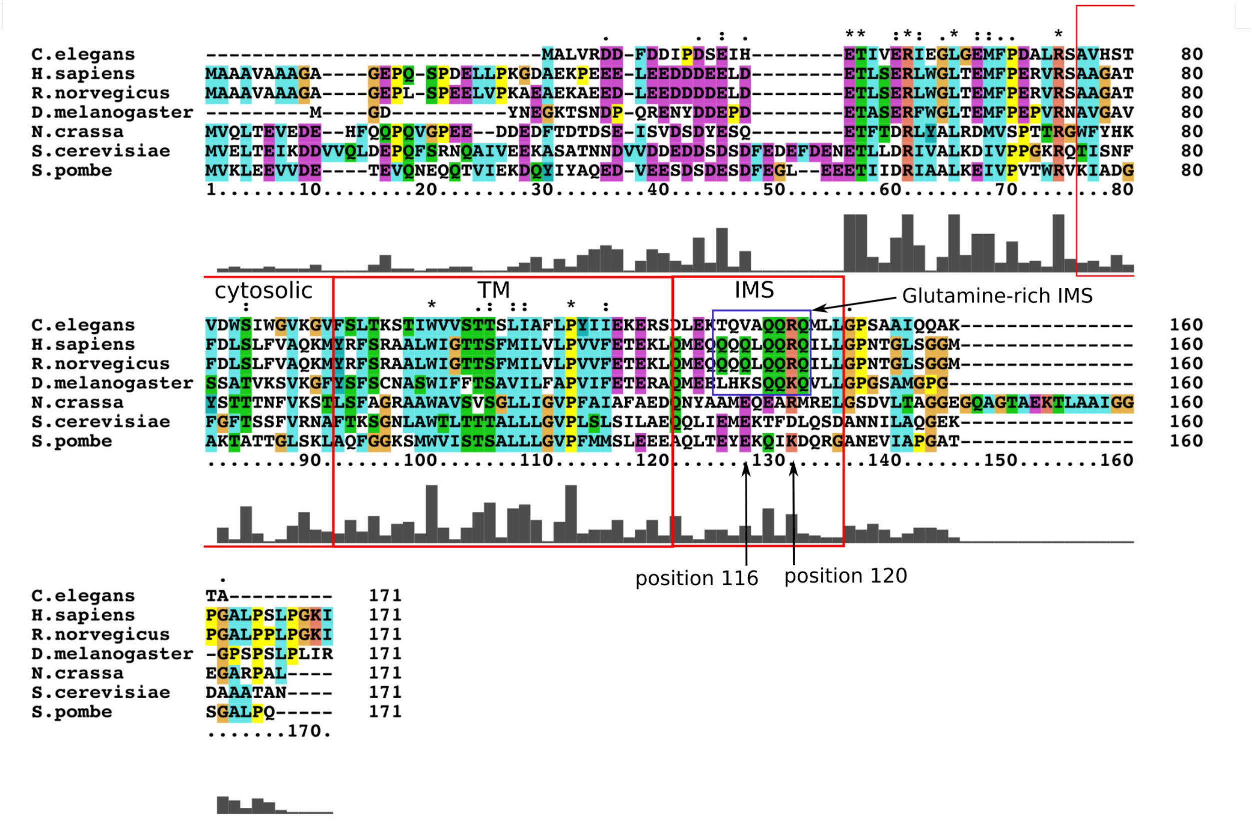

Figure S3: Multiple sequence alignment of Tom22 protein sequences from fungal and mammalian species. The boxes mark the helical region of Tom22 with the distinct cytosolic, transmembrane (TM) and intermembrane space (IMS) segments, whereas the rest of the region is either disordered or currently unresolved in the available structures. The conserved Proline residue within the TM segment marks the helix kink.

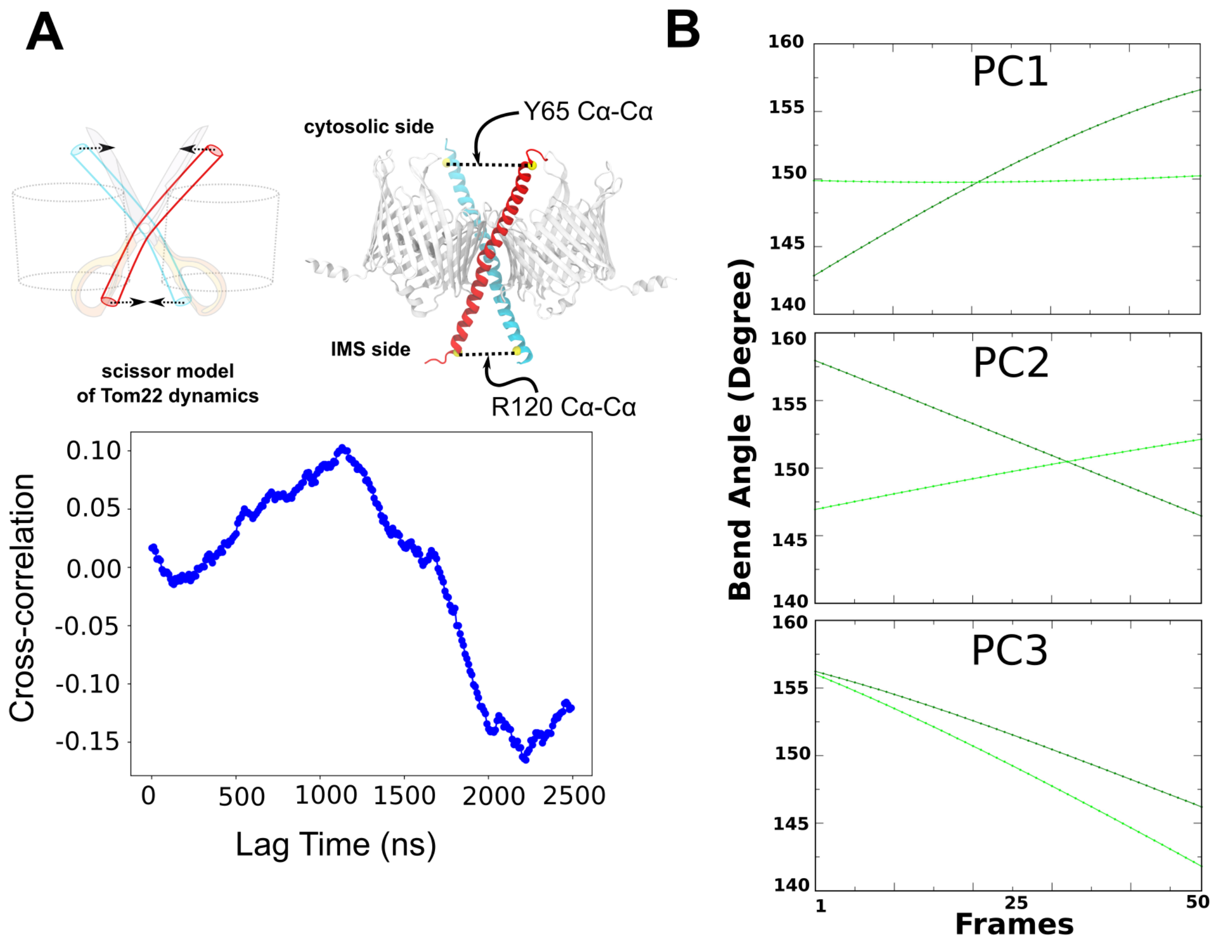

Figure S4: The dynamics of Tom22 helices does not constitute a scissor-like motion. (A) The scissor model of Tom22 dynamics would assume that the Y65 Cα-Cα and R120 Cα-Cα distance are linearly correlated. A plot of the cross-correlation between the projections on the xy-plane of Y65 Cα-Cα and R120 Cα-Cα distances at different lag times, indicating no significant linear relationship. (B) A plot of the change in Tom22 bend angle along the top three eigenvectors calculated from the PCA analysis of TOM22. The two lines represent the change in the bend angle for the two Tom22 helices. Residues 65 to 122 were selected to exclude the highly disordered terminal segments. Before the PCA step, the helices were aligned at the hinge region (residues 97–100), where minimal relative motion occurs between the two helices. Note that the bend angle change has been calculated from a trajectory obtained through a smooth interpolation between the two extreme projections on the helix along the eigenvectors.

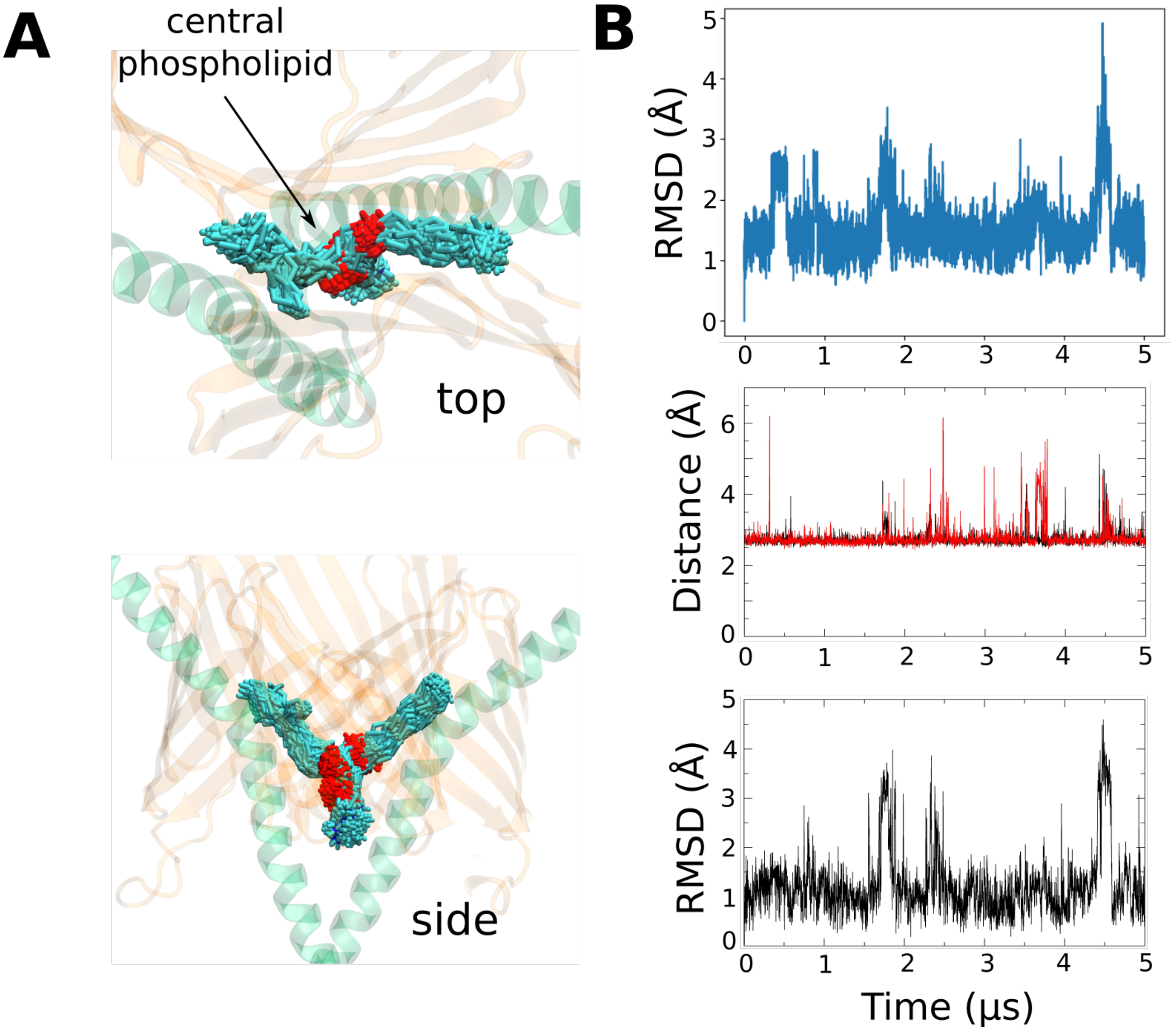

Figure S5: Conformational fluctuations of the central phospholipid molecule. (A) The central lipid shows conformational fluctuations in the acyl chains. The lipid conformations shown here were extracted from a representative 5 microseconds-long unbiased simulation trajectory. (B) The conformational fluctuations in the lipid was quantified as the rmsd of the lipid heavy atoms (top panel). The middle panel plots the distance between the oxygen atoms of the phosphate group and the interacting K298 residue supplied by the two Tom40 barrels. The bottom panel plots the RMSD of the glycerol backbone of the lipid molecule.

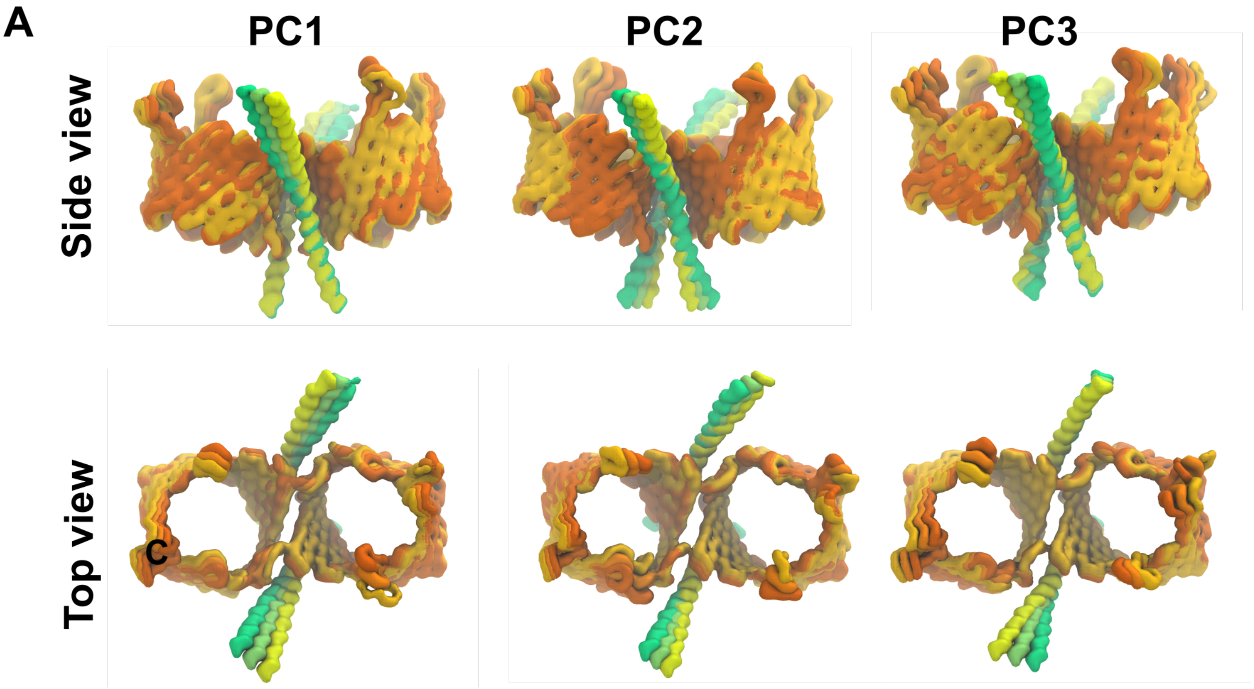

Figure S6: PCA analysis of Tom40 and Tom22 motions. The dominant motions of Tom22 and Tom40 are described by the top principal eigenvectors calculated from the principal component analysis of the Cα atoms of Tom22 and Tom40 chain. The extreme projections of the Tom40 are depicted in shades of orange and that of Tom22 in shades of green. Also see the supplementary video 1.

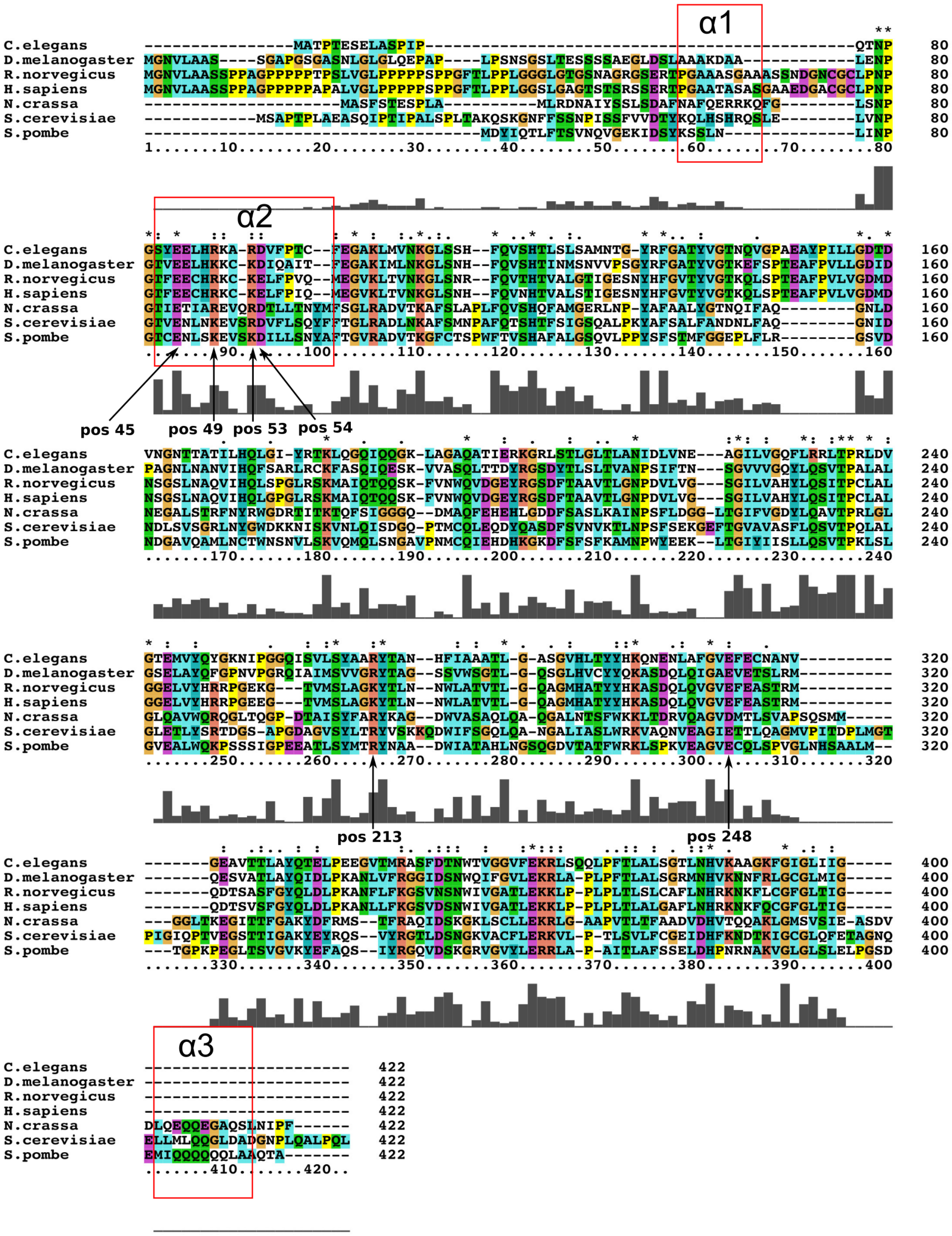

Figure S7: Multiple sequence alignment of Tom40 sequences from fungal and mammalian species. The boxes mark the helical regions in the N-terminal ($\alpha 1$ and $\alpha 2$) and C-terminal ($\alpha 3$).

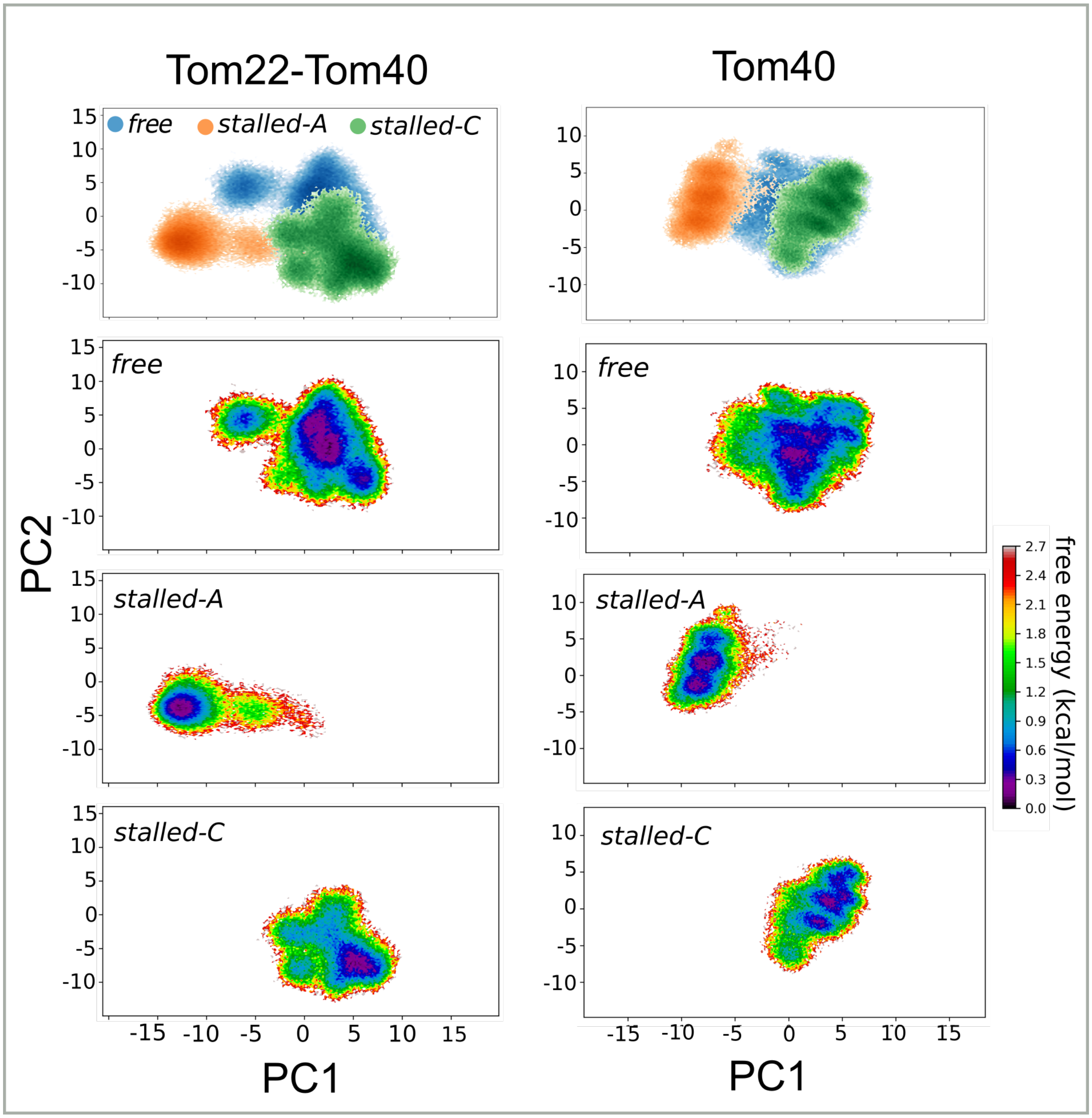

Figure S8: Principal component analysis of the *free*, *2ptA* and *2ptC* simulations. PCA was performed on the combined trajectories of the three datasets, and the backbone phi (ϕ) and psi (ψ) angles of Tom40 and Tom22 (left column), and Tom40 alone (right column) chosen as the input for the dihedral PCA. The top panels plot the PCA projects of the different simulations along the PC1-PC2 space. The lower panels show the corresponding free energy for the individual datasets.

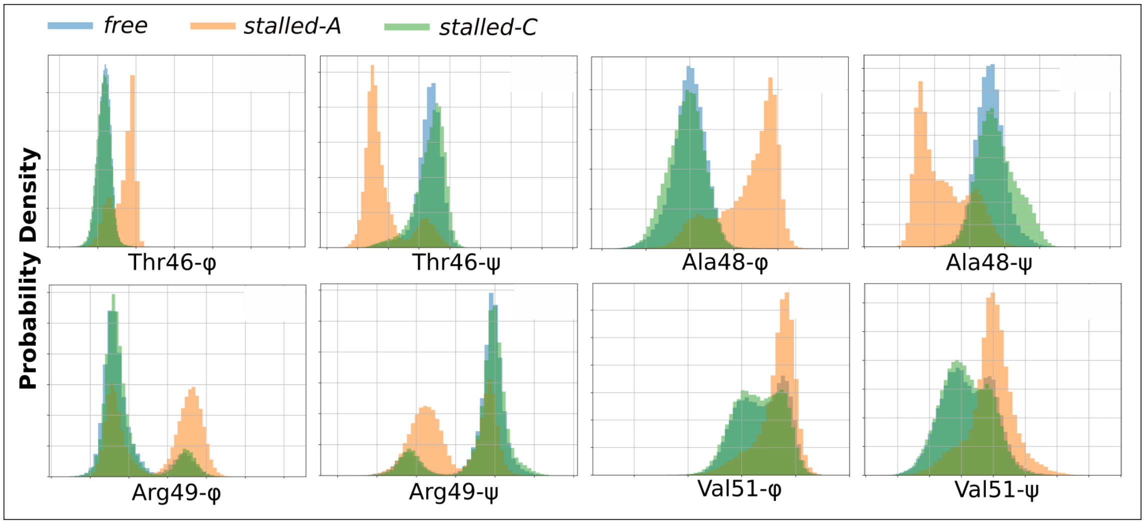

Figure S9: Probability densities of the backbone Ramachandran angle (ϕ and ψ) of the residues in α2 helix as observed in the *free*, *stalled-A* and *stalled-C* simulations. The stalled-A simulations show significant deviations attributed to the α2-P to α2-F transition. The x and y axes use an arbitrary range to focus only on the density shifts between. The axes values have therefore been omitted.

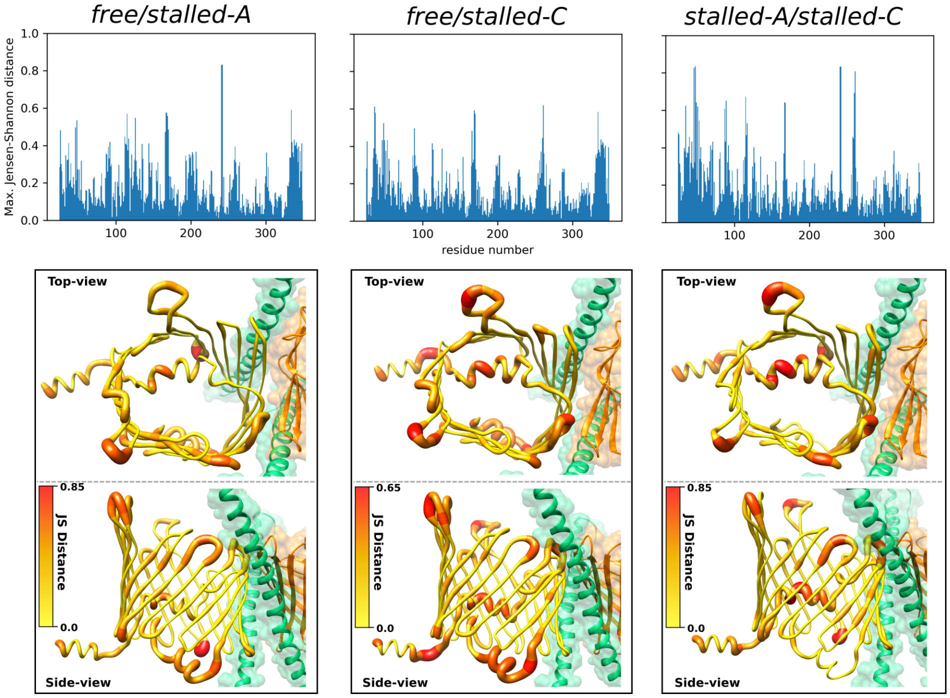

Figure S10: Jensen-Shannon distance (JSD) between the backbone torsional distribution extracted for a pair of ensembles among the *free*, *stalled-A* and *stalled-C* datasets. Results for the three possible pairs ***free/stalled-A*** (left), ***free/stalled-C*** (center) and ***stalled-A/stalled-C*** (right) are depicted. The top panels plot the per-residue JSD for the residues in Tom40. The bottom panels convey the same information mapped onto the Tom40 structure with the JSD information encoded using the color and thickness of the ribbons. The Tom22 helices at the dimer interface is shown in green color.

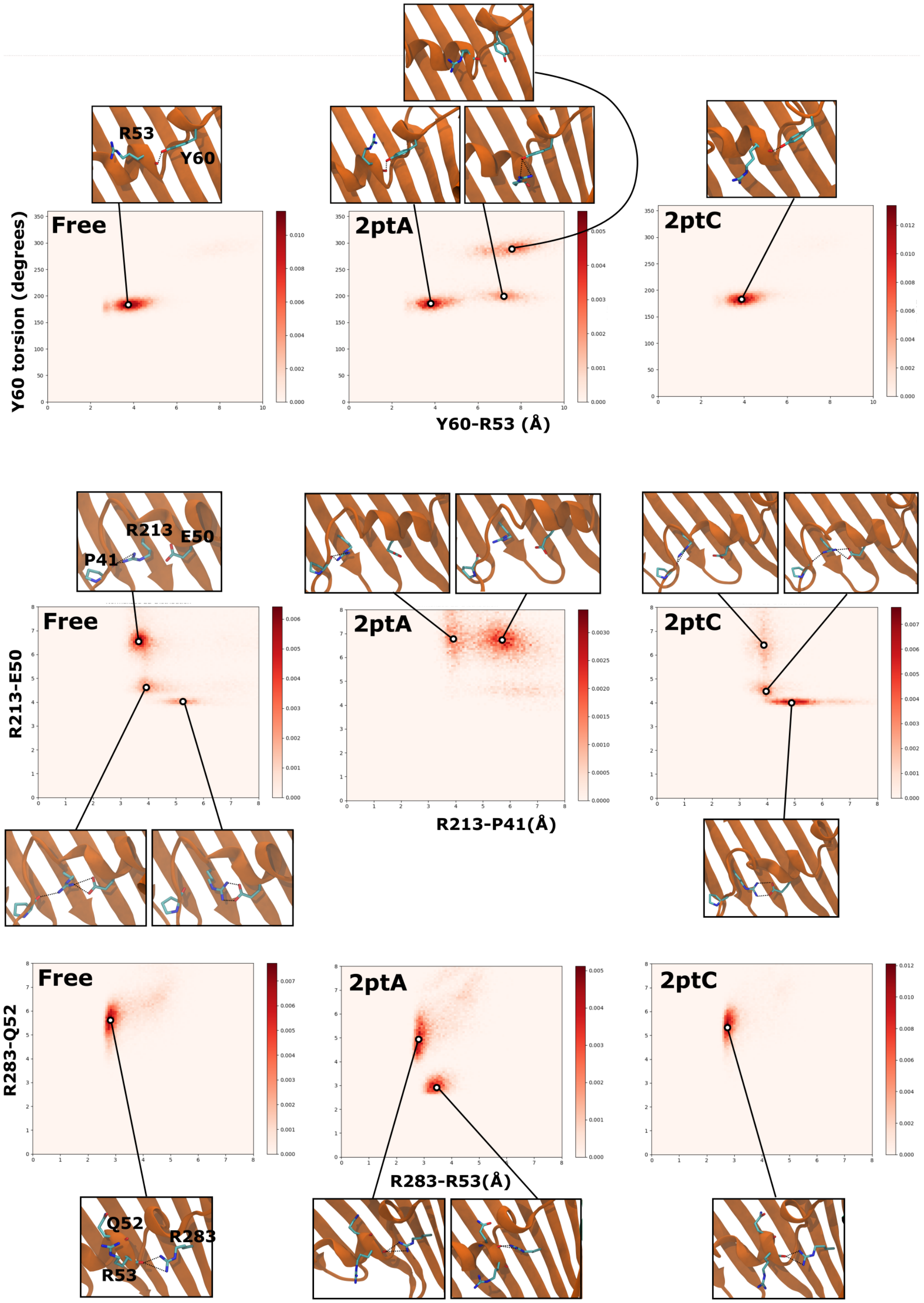

Figure S11: Important residues that undergo significant changes in their dynamics and interactions between the *free* (column 1), *stalled-A* (column 2) and *stalled-C* (column 3) simulation sets. The plots show the distribution of the distance between residues interacting through hydrogen bonds. For the first row, the y-axis is the $\chi1$ torsion of the Y60 residue. The representative conformation corresponding to the clusters are also depicted.

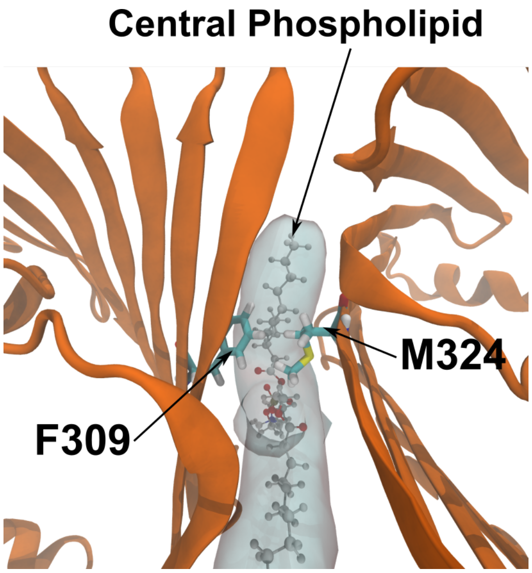

Figure S12: The F309-M324 interaction at the dimer interface of the Tom40 pores. This interaction is a prominent one in the network analysis with a high betweenness centrality (see Table S3 and S12).

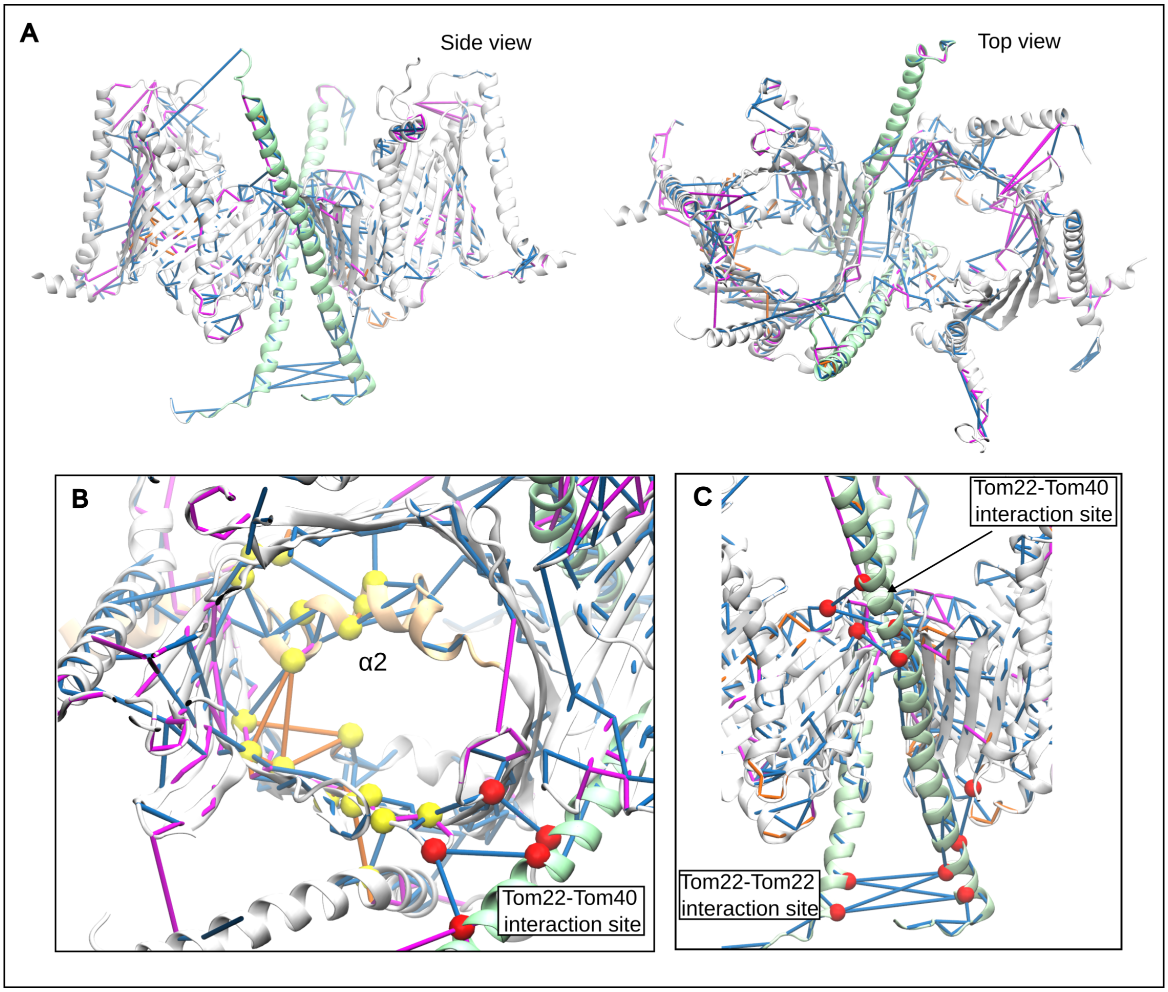

Figure S13: Force distribution upon Tom22 stabilization during stalling. (A) The network of force differences between the free and 2PTA simulations is shown as sticks. Only connected graphs with more than 5 residues are shown for clarity. The side and top views of the TOM complex are shown. The different colors of sticks indicate different force cutoffs of 75 pN (blue), 150 pN (magenta), and 250 pN (orange). (B) The residues with the highest total force difference that are involved in inter-domain interactions between Tom22 and Tom40 subunits are depicted as red beads. (C) The residues with the highest total force difference within the Tom40 barrel are depicted in yellow beads.

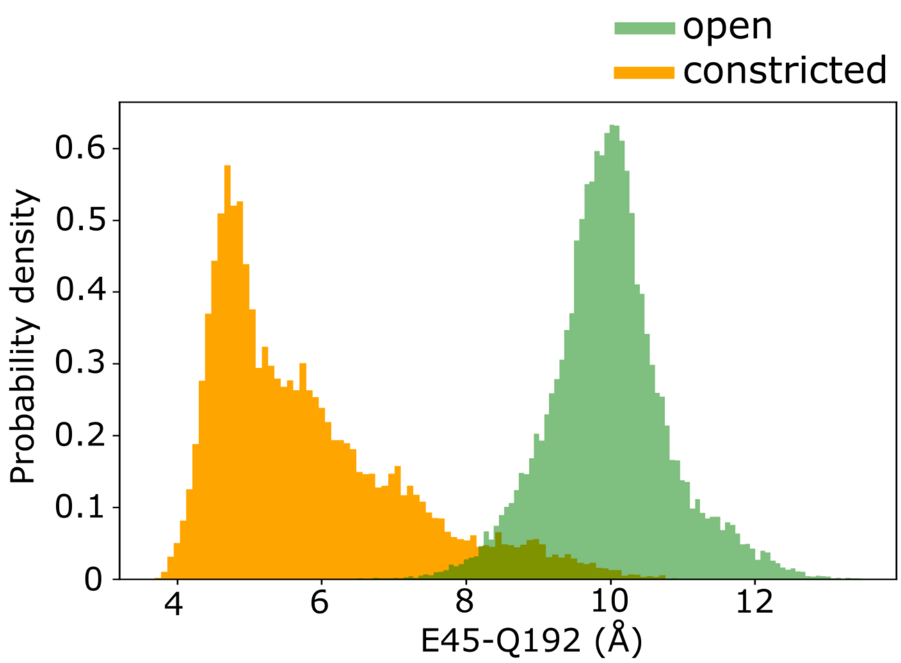

Figure S14: The distance distribution for the E45-Q192 interaction extracted from simulations of the open and constricted TOM complexes.

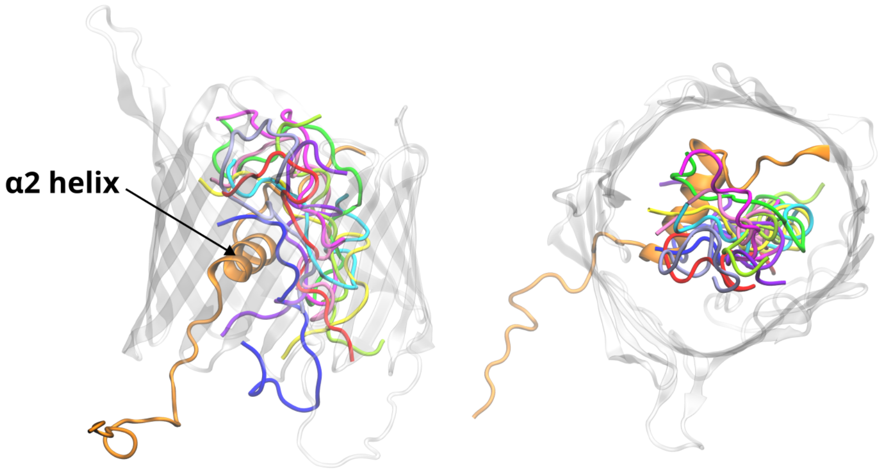

Figure S15: Configurations of the first 19 amino acids of the precursor peptide of rat dehydrogenase inside the lumen of the constricted state of Tom40 obtained from the stalled simulations. These configurations were generated using the Rosetta modelling suite. We show here 10 representative peptide configurations traversing the Tom40 lumen.
